# Regional heterogeneity in coral species richness and hue reveals novel global predictors of reef fish intra-family diversity

**DOI:** 10.1038/s41598-021-97862-8

**Published:** 2021-09-14

**Authors:** Kieran D. Cox, Mackenzie B. Woods, Thomas E. Reimchen

**Affiliations:** 1 Department of Biology, University of Victoria, Cunningham 202, 3800 Finnerty Road, Victoria, BC V8P 5C2 Canada; 2 Hakai Institute, Heriot Bay, BC V0P 1H0 Canada

**Keywords:** Marine biology, Biodiversity, Community ecology

## Abstract

Habitat heterogeneity shapes biological communities, a well-known process in terrestrial ecosystems but substantially unresolved within coral reef ecosystems. We investigated the extent to which coral richness predicts intra-family fish richness, while simultaneously integrating a striking aspect of reef ecosystems—coral hue. To do so, we quantified the coral richness, coral hue diversity, and species richness within 25 fish families in 74 global ecoregions. We then expanded this to an analysis of all reef fishes (4465 species). Considering coral bleaching as a natural experiment, we subsequently examined hue's contribution to fish communities. Coral species and hue diversity significantly predict each family's fish richness, with the highest correlations (> 80%) occurring in damselfish, butterflyfish, emperors and rabbitfish, lower (60–80%) in substrate-bound and mid-water taxa such as blennies, seahorses, and parrotfish, and lowest (40–60%) in sharks, morays, grunts and triggerfish. The observed trends persisted globally. Coral bleaching's homogenization of reef colouration revealed hue’s contribution to maintaining fish richness, abundance, and recruit survivorship. We propose that each additional coral species and associated hue provide added ecological opportunities (e.g. camouflage, background contrast for intraspecific display), facilitating the evolution and co-existence of diverse fish assemblages.

## Introduction

Habitat heterogeneity is widely recognized as a universal driver of species diversity that spans taxa, ecological communities, and biomes^1^. As a cornerstone concept in ecology, diverse habitats promote speciation events, and support biodiverse communities through increasing available niche space, providing refugia from adverse conditions, and offering a range of resources for species to exploit^1,2^. The role of habitat heterogeneity in facilitating diverse ecological communities has been observed across taxa, including birds, reptiles, mammals, insects, aquatic invertebrates, and is perhaps the most evident within coral reef fish^2–7^. While structural heterogeneity in a habitat is a widely recognized framework for niche diversity and ecological opportunity, hue can also shape the perception of biological processes such as social signaling, mimicry, and aposematism^8,9^. This causes colouration to mediate the visual relationship between an organism and its environment^9^. Consequently, predation and other visually mediated selective pressures will favor organisms that operate in accordance with the background colouration^8,10–12^. As ecological processes occur over a range of distances, the interplay between a species colour, pattern, and background colouration is a critical aspect of ecosystems dynamics, with the interaction between these covariates influencing population persistence^8,9,12–15^.

Marine ecosystems globally support 20,000 fish species, of which 30% occur in association with the geographically restricted coral reefs of equatorial zones. Among these reefs, fish species diversity ranges from low levels in the Atlantic to high levels in the west Pacific, a geographical trend that broadly parallels the abundance of coral species. This covariation is associated with habitat complexity and subsequent niche partitioning provided by diverse coral communities^3,16,17^. Spatial use of the reef can be specialized as in the case of numerous seahorses (Syngnathidae) that are restricted to several species of Gorgonian coral, through to spatial generalists such as barracuda (Sphyraenidae) and jacks (Carangidae) that move across the entire reef^18–21^. Substrate-bound taxa such as gobies (Gobiidae) and blennies (Blenniidae) are species rich and associated with spatial heterogeneity on the reefs^22^. Despite the major research on reef fishes^23–26^ the relationships between intra-family species diversity and coral diversity within and among global oceanic basins has not been quantified.

A principal ecological attribute of coral reef communities is their spectral variability. This comprises the widely-recognized diversity of colours and patterns that vary ontogenetically within and among fish species^14,27,28^. It also includes the hue diversity imparted by scleractinian corals. Produced by varying concentrations of host-based fluorescent proteins and endosymbiotic dinoflagellates pigments^29,30^, coral hue contributes a kaleidoscope of colours to the ecological backdrop^9,14,31^. The heterogeneous distributions of coral species globally, combined with the various configurations of con- and hetero-specific corals within any particular region, causes colour diversity to vary considerably across coral reef ecosystems^30,32,33^. However, the connection between taxonomic richness and habitat hue is unsubstantiated within global reef communities. This limitation has endured despite the recognition that coral reef teleost fish exhibit mono and penta-chromacy, and perceive, albeit at different sensitivities, the colouration of corals^33–37^. At short distances, fish colour vision functions similarly to terrestrial equivalents, with pigmentation being an informative component of perception, but as distances increase, detection of visual contrasts relative to background colouration becomes increasingly important^12,14,15,33^. As fishes interact amongst varying coral communities and over a range of distances, the interplay between fish communities and coral colouration may represent a critical aspect of coral reef ecosystems^12,14,33^.

In this paper, we examine the extent to which coral species richness predicts fish species richness within each of the 25 most common fish families found on reefs for 74 ecoregions across Pacific, Indian and Atlantic oceans^26,38^. Secondly, we apply a novel method of quantifying digital image colouration to Corals of the World’s 784 coral species images and enumerated hue diversity within each of the 74 ecoregions^38,39^. We then integrated these data into the analyses of fish and coral taxonomic diversity by evaluating coral hue diversity as a covariate facilitating fish diversity, which may function concurrently or independently of coral species richness^40^. We then expanded this analysis to examine the contribution of the relationship observed within the 25 common reef fish families to maintaining global reef fish diversity. To do so, we evaluated coral richness and hue diversity as separate and combined predictors of the total reef fish diversity (4465 species). Finally, a subsequent examination considered coral colour loss as a natural experiment examined reef hue's contribution to maintaining fish diversity, abundance, and recruit survivorship. We predict that the number of small substrate-bound fish species (e.g. seahorses-Syngnathidae, gobies- Gobiidae) will have the highest positive associations with number of coral species, large-bodied and mobile herbivorous species (e.g. parrotfish- Scarini (formerly Scaridae), surgeonfish- Acanthuridae) lower associations and larger-bodied and mobile predators (e.g. sharks- Carcharhinidae, jacks- Carangidae) the lowest associations. Furthermore, we postulate that integrating coral hue diversity as a covariate predictor of fish richness will explain additional variability, a trend that will persist within and among fish families, and across oceanic basins.

This examination represents the first multivariate analysis of coral species and hue diversity's contribution to reef fish intra-family diversity. Geographic and taxonomic consistency in the trends would suggest that coral hue diversity is an important ecological mechanism that functions in combination with coral species richness to facilitate the evolution and co-existence of diverse coral reef fish assemblages. Our consideration of coral bleaching may elucidate whether the spectral diversity of coral cover, independent of coral species diversity and structural complexity, is a contributing covariate facilitating fish diversity, abundance, and recruit survivorship. By examining coral species and hue diversity's contribution to reef fish diversity across geographical regions and within a diverse array of fish families, we aim to focus new attention on the ecological importance of the kaleidoscope of colours exhibited by healthy coral reefs in shaping the perception of visually mediated selective pressures.

## Results

### Coral richness, hue, and intra-family fish richness

**Figure 1:**
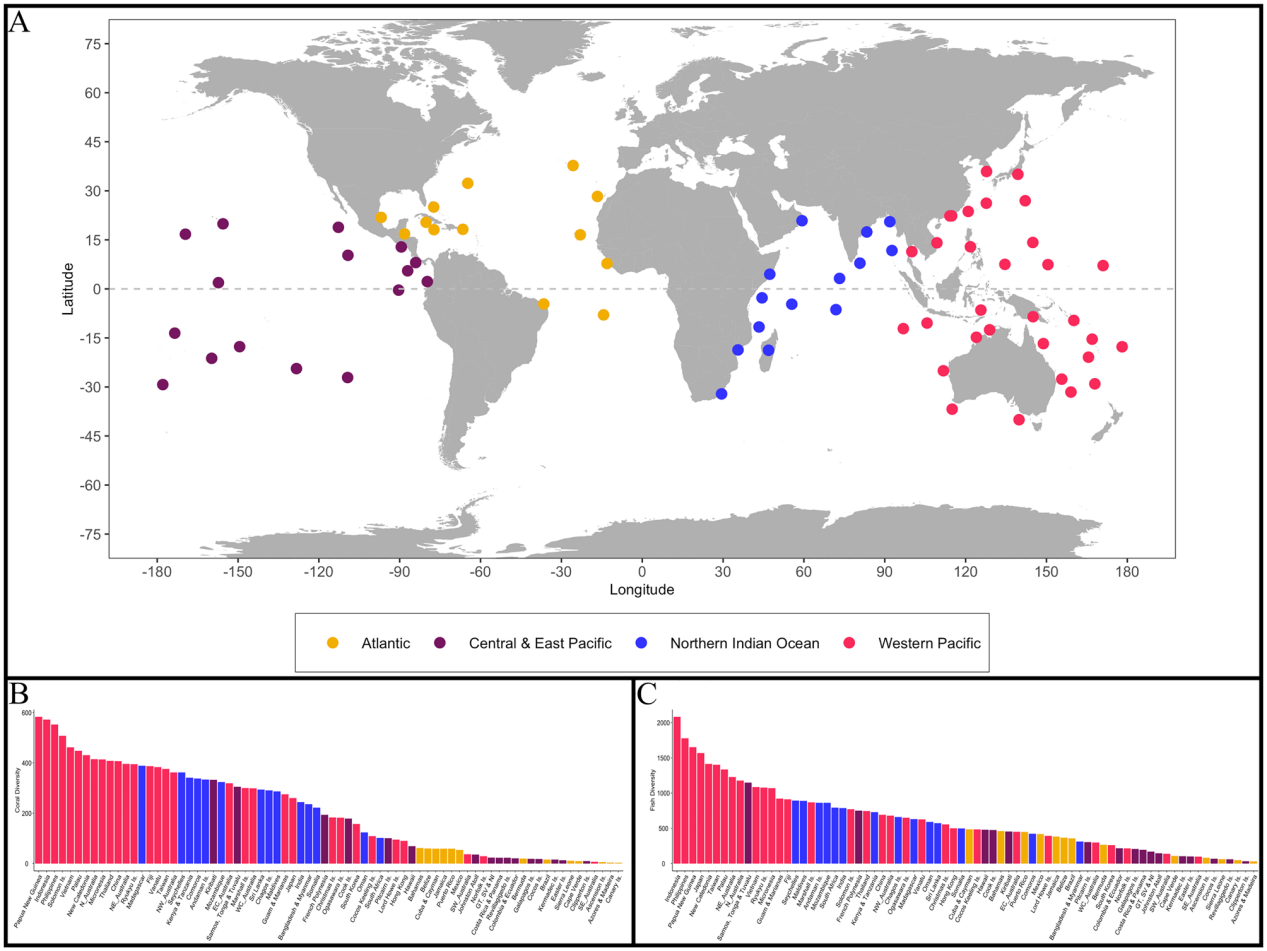
(**A**) 74 global ecoregions categorized into four oceanic regions, represented by distinct colours (**B**) Coral species richness present in each ecoregion (**C**) Fish species richness present in each ecoregion. Generated using the ‘ggplot2’ package in RStudio version 3.6.1^41^. Figure 1A was created using ggplot2’s map_data function.

**Figure 2:**
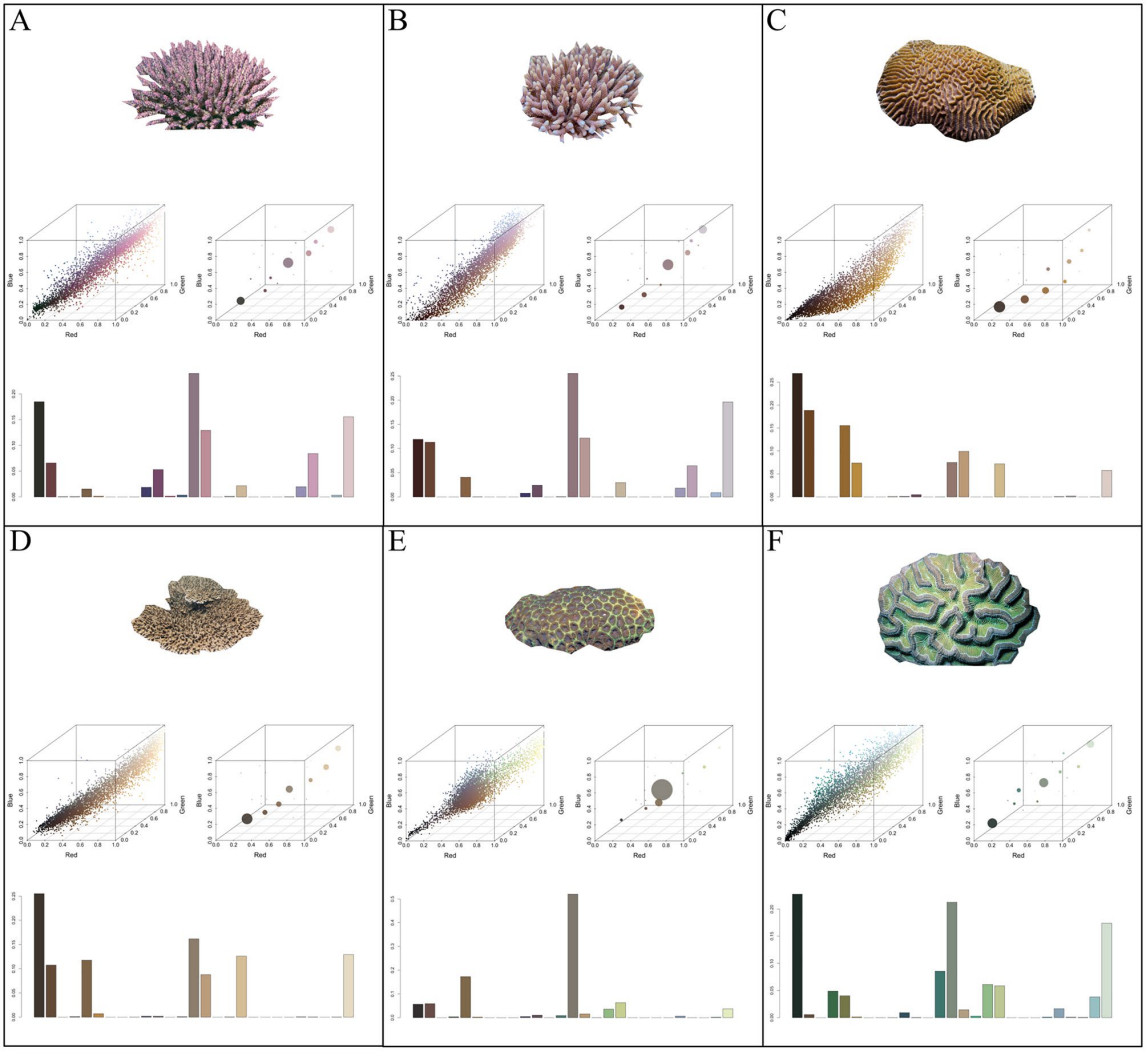
Example of coral hue quantification. Illustrates the cropped coral image, hue profiles in RGB colour space, and the resulting hue histogram bins. (**A**) *Acropora subulata* (**B**) *Acropora millepora* (**C**) *Platygyra acuta* (**D**) *Acropora solitaryensis* (**E**) *Favites bestae* (**F**) *Colpophyllia natans*.

**Figure 3:**
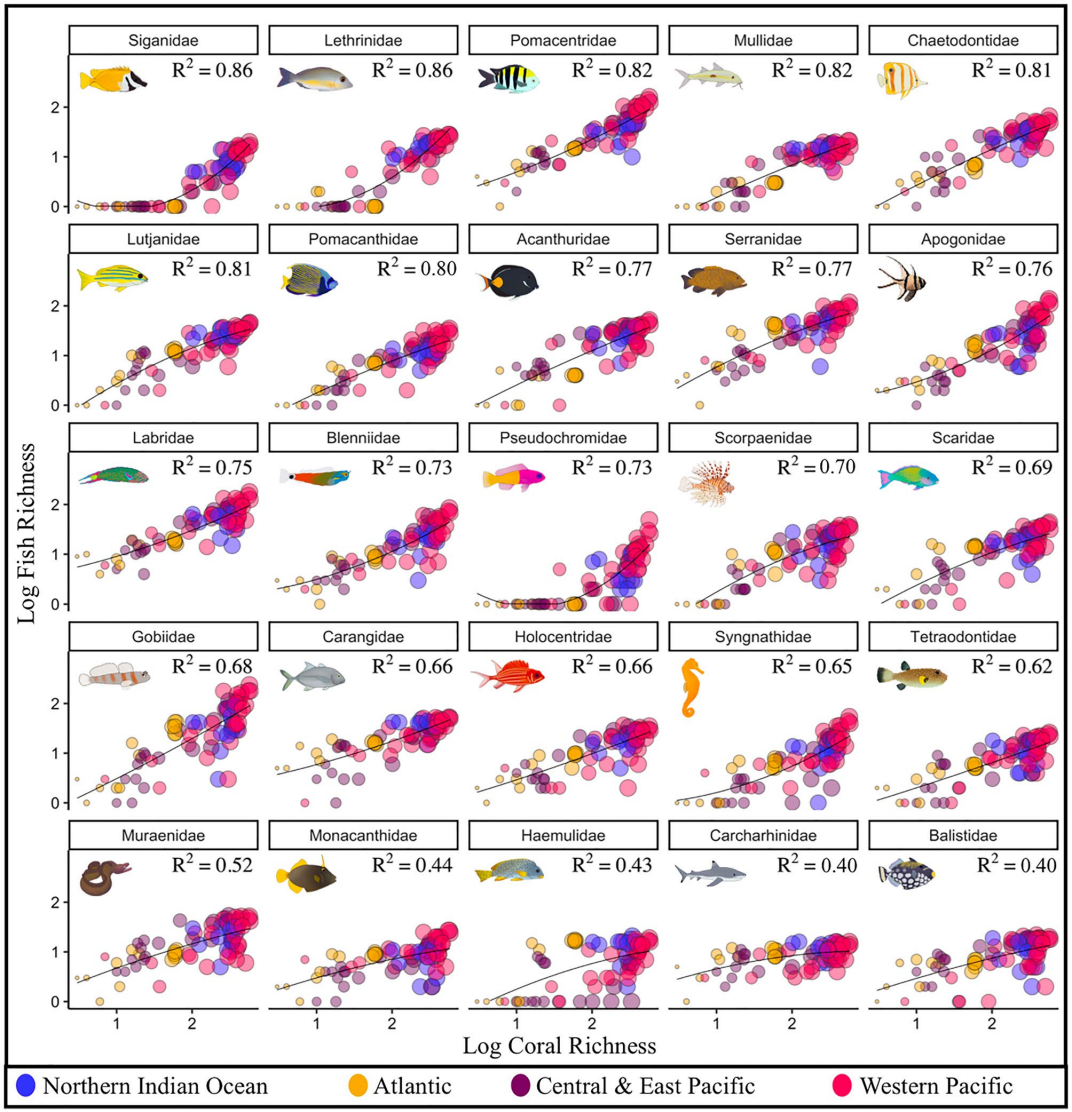
The relationship between fish intra-family richness, coral species richness, and coral hue diversity, within 25 common reef-associated fish families in each of the 74 ecoregions. Each point denotes an ecoregion, and oceanic regions are represented by distinct colours. The size of each circle is proportional to the hue diversity within each ecoregion. Illustrations show a representative species from each family. Families ordered by r-squared value.

Coral species, associated hue diversity, and reef fish richness were all the highest within the Western Pacific and declined with increasing distance from this region (Figs. 1, 2, S3, Table S1). Within all 25 common reef fish families, species richness was positively correlated to coral richness and hue diversity (Figs. 3, S8, S9). The strength of these relationships, however, varied among families (Fig. 3). Coral richness and hue diversity explained 40–60% of the variance in large-bodied midwater fish families, including Carcharhinidae (requiem sharks) and Carangidae (e.g. jacks, mackerels). Between 60 and 70% of the variability in species richness within families that exhibit tightly coupled coral-fish relationships (e.g. mutualism, commensalism, Blenniidae, Gobiidae, Scorpaenidae) was explained by coral richness and hue diversity (Fig. 3). Generally, substrate-bound fish families exhibited the greatest association with coral richness and hue diversity. Within Siganidae (rabbitfish), Lethrinidae (emperors), Pomacentridae (damselfishes), Mullidae (goatfish), Chaetodontidae (butterflyfish), Lutjanidae (snappers), and Pomacanthidae (angelfish), coral richness and hue diversity predicted more than 80% of the variance in fish richness. Among the 25 families, hue's inclusion in this three-way interaction increased the variance explained in fish richness from 0 to 9% relative to when only coral richness was considered (Figs. 3, S8, S9). These patterns varied considerably among the four oceanic regions (Figs. S8, S9). Specifically, the families with the most explained variability, and the extent to which coral richness and hue diversity predicted fish intra-family richness, varied among the oceanic regions. The variation in fish diversity explained ranged from 40 to 86% when coral diversity and hue were analyzed concurrently, and from 33 to 86% and 37 to 86% when the analysis considered coral hue and coral diversity separately (Figs. 3, S8, S9, S10).

### Coral richness, hue, and global reef fish richness

**Figure 4:**
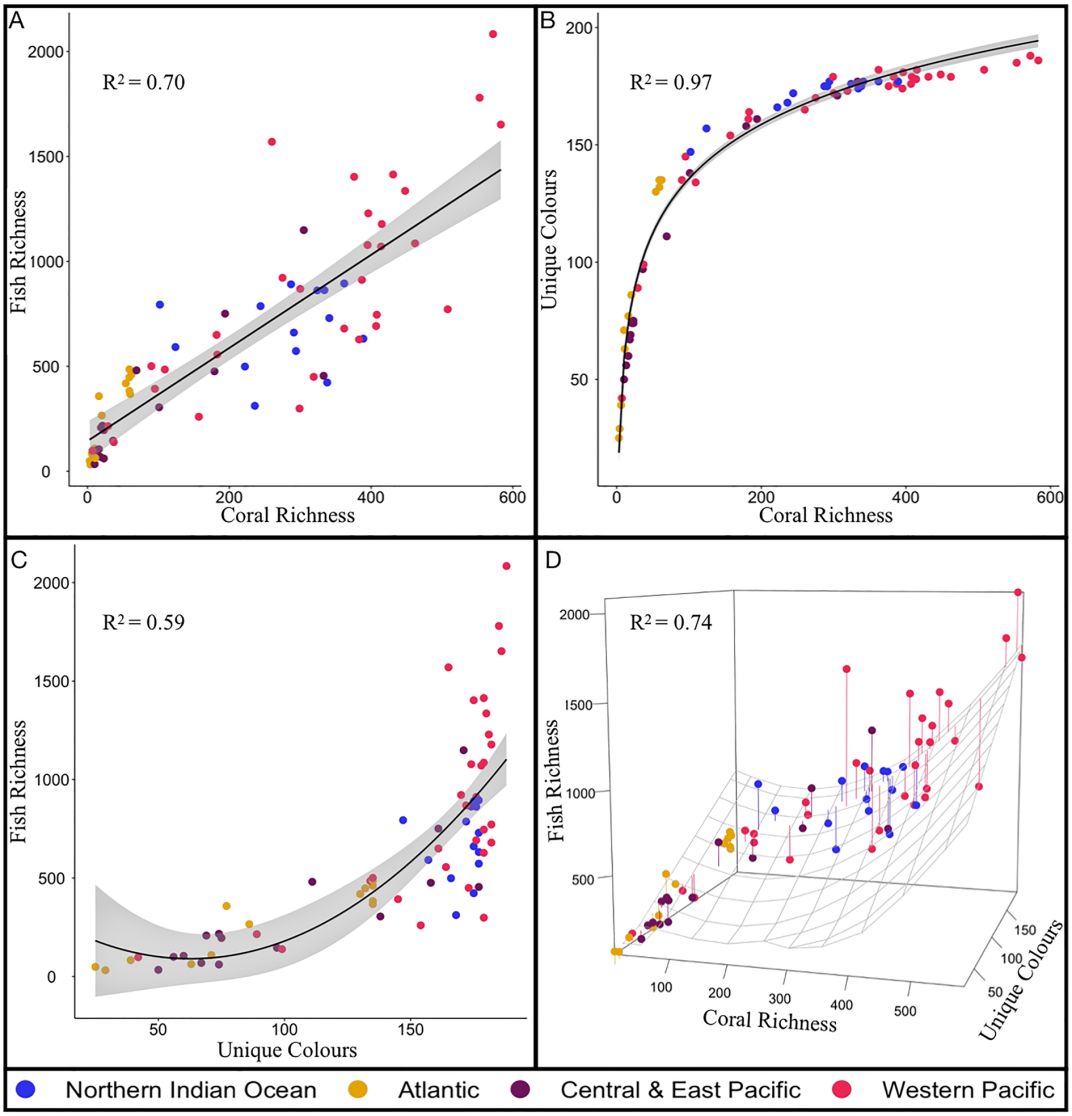
The relationship between 784 coral species, 4465 fish species, and coral hue across 74 ecoregions. Each point denotes an ecoregion, and oceanic regions are represented by distinct colours. (**A**) Linear model of the reef fish and coral richness present in each ecoregion. (**B**) Logistic regression of the number of corals richness and associated hue diversity in each ecoregion (**C**) Quadratic (second-order) polynomial model of the unique hue diversity and fish richness within each ecoregion (**D**) Multiple regression evaluating the influence of hue diversity, and coral richness as a quadratic (second-order) polynomial term, on reef fish richness (**A**–**D**) Adjusted R-Squared values reported (**A**–**C**) Standard error illustrated in grey.

Taxonomic associations could be attributed to geographical or classifications biases; therefore, we examined coral richness and hue diversity, separately and in combination, as predictors of 4465 reef fish species from 117 fish families within the 74 global ecoregions. Across all regions, fish richness was linearly correlated to coral richness (Adjusted R^2^ = 0.70, F (1, 72) = 169.9, *p* < 0.001) and exponentially correlated to hue diversity (Adjusted R^2^ = 0.59, F (2, 71) = 54.3, *p* < 0. 0.001; Fig. 4). The diversity of coral hues increased with coral species, plateauing at approximately 180 unique colours and a minimum of 300 species of coral (Fig. 4; Adjusted R^2^ = 0.97, F (1, 72) = 2633, *p* < 0.001). In combination, the richness of corals and associated hues were positively correlated with fish richness (Adjusted R^2^ = 0.74, F (3, 70) = 69.15, *p* < 0.001), with the combination of both accounting for more of fish community’s variability than when either was evaluated separately (Fig. 4). Furthermore, model selection supported coral richness and associated hue as the best predictor of fish richness (Table S2). The influence of coral richness and hue on fish intra-family richness varied considerably among the four oceanic regions (Figs. S7, S8, S10). The Central and East Pacific (Adjusted R^2^ = 0.75, F (3, 12) = 16.3, *p* < 0.001), and Western Pacific (Adjusted R^2^ = 0.60, F (3, 27) = 16.3, *p* < 0.001.) exemplified the global trend (Fig S7). Whereas, coral richness and hue diversity's correlation with reef fish richness was considerably higher within the Atlantic relative to the global average (Adjusted R^2^ = 0.88, F (3, 9) = 30.8, *p* < 0.001; Fig S7). The extent of this association was reduced within the Northern Indian Ocean due to higher fish richness within South Africa and Oman (Adjusted R^2^ = 0.09, F (4, 9) = 1.31, *p* > 0.1; Fig S7).

### Coral hue homogenization

**Figure 5:**
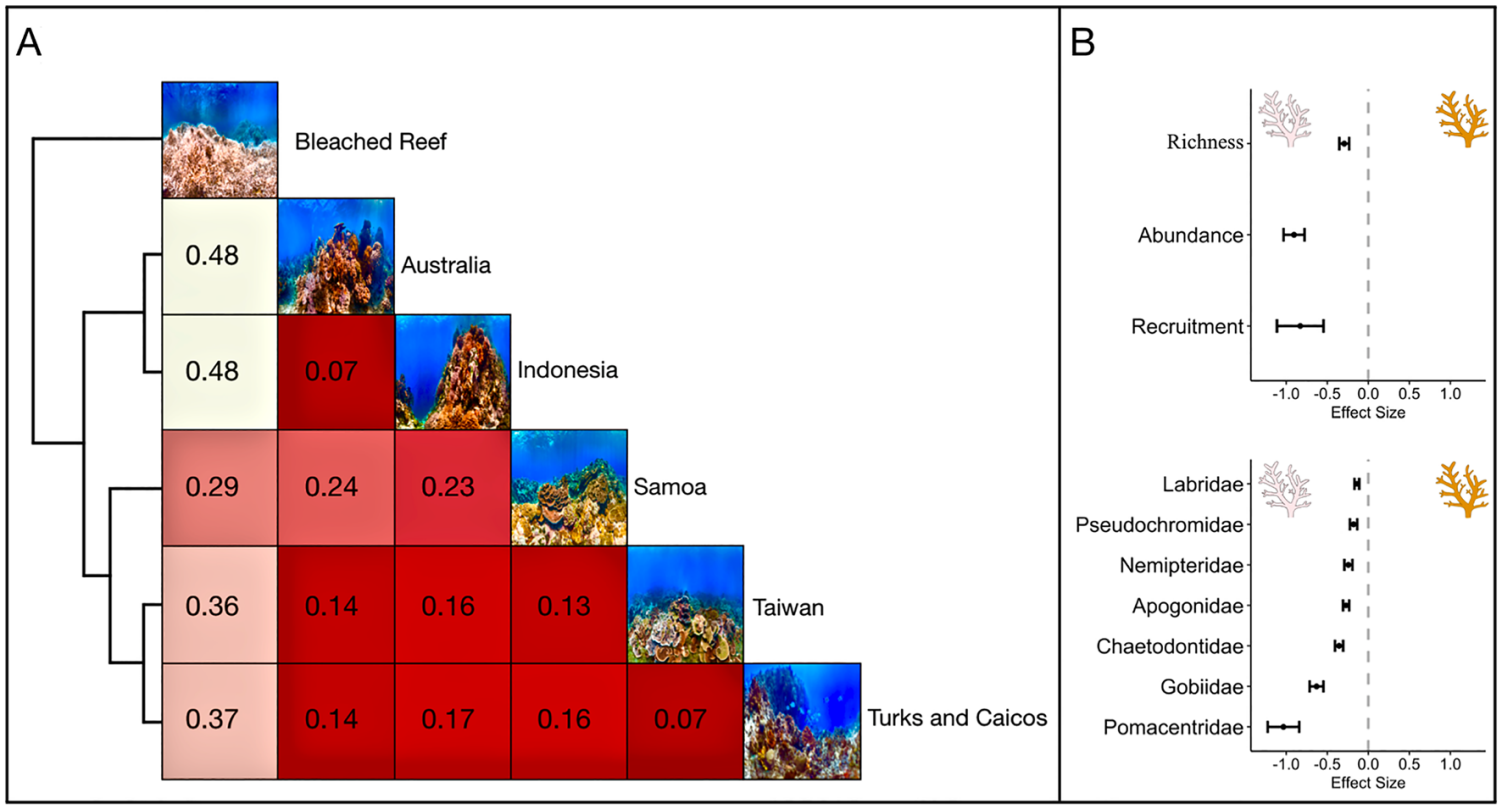
(**A**) A distance matrix evaluation of reef colour similarity between Ocean Agency images of American Samoa (Fogama), Australia (Lizard Island), Indonesia (Manado), Taiwan (Donghi Harbor), Turks and Caicos (Providenciales), and a severely bleached reef surveyed in American Samoa collected during the Catlin Seaview Survey retrieved from Coral Reef Image Bank^42^. A heatmap plot illustrates the resulting distance matrix, clustered by similarity. Colour and number in boxes denotes pairwise dissimilarity between comparisons (e.g. higher numbers and whiter squares indicate more colour dissimilarity between comparisons). (**B**) An analysis of published literature evaluating reef fish responses to coral bleaching events during which coral richness and structure were maintained. Effect sizes and standard error shown.

Reefs exhibit distinct hue heterogeneity. The colour distance matrix arranged the bleached and healthy reefs according to observed hue diversity, forming three separate groups, which primarily corresponded to the region’s coral richness (Figs. 1, 5). The Australian and Indonesian reefs formed a distinct group relative to the other areas considered, indicating that their colouration is similar. Taiwan, and Turks and Caicos exhibited a comparable orientation; however, their colouration was more analogous to American Samoa than to Australia or Indonesia. Coral bleaching homogenized colouration, producing the most substantial measure of colour dissimilarity (Fig. 5). Among the pairwise distance comparisons of the bleached and healthy reefs, elevated levels of dissimilarity corresponded to increased colour diversity within the unbleached reefs. An analysis of the published literature indicated that coral bleaching-induced colour homogenization decreases fish species richness, abundance, and the survivorship of recruits, with declines in abundance varying among fish families (Fig. 5, Table S3).

## Discussion

We establish for the first time the extent to which coral richness and hue diversity, when considered separately and in combination, predict fish species richness within each of the 25 common families found on reefs. We then determined this bivariate relationship persists among 4465 reef fish species present across the 74 ecoregions. These findings collectively suggest coral hue diversity may be an unrecognized ecological mechanism supporting taxonomic diversity within and among reef fish families. The species richness within fish communities expands exponentially with increasing hue diversity, conceivably supported by ancillary biological processes that are sustained within a more diversely coloured seascape. Furthermore, the richness of a reef’s fish community correlates linearly with increasing coral species richness, implying the two biological communities are ecologically linked. Collectively, the striking relationship between the species richness and hue diversity of scleractinian corals and associated reef fish is replicated among fish families and across geographical regions. Consequently, coral richness and colouration predict fish communities globally, with fish richness varying according to a region's unique coral richness and associated colouration. Replicating our analysis across geographical regions accounts for variability in the ecological processes that govern reef ecosystems at local and regional scales, including reef area, environmental conditions, reef isolation, metapopulation dynamics, and reef geological age^17,19,21,43,44^. The consistent correlation between coral richness, hue diversity, and fish intra-family richness indicates this relationship structures reef fish communities among and within fish families and extends beyond taxonomic biases.

The most parsimonious model contained both coral richness and coral hue as predictor variables. The additional variability described under this condition indicates coral richness’ and hue diversity’s contributions to structuring reef fish communities are not equivalent, and that examining the variables in combination is necessary to effectively describe fish assemblages. This distinct relationship persists within a diverse array of fish families. Furthermore, the order of the 25 families, when ranked by how much intra-family variability was explained by the model, differed depending on whether coral richness and hue diversity were considered separately or in combination. This further supports the validity of considering coral richness and hue diversity concurrently when describing reef fish communities. Consequently, diverse reef fish assemblages are limited to regions with coral richness and colouration rich enough to facilitate the ecological processes needed to support these communities. Deviations from this relationship can be attributed to both biotic and abiotic conditions that disrupt species-habitat associations. For example, South Africa and Japan exhibited elevated fish richness relative to coral species richness and hue diversity. However, South Africa and Japan experience cooler water temperatures than surrounding ecoregions. This condition encourages the influx of cold-water tolerant fish species but excludes less tolerant coral species^45,46^.

Coral richness and associated hue diversity provide an ecological backdrop that shapes the perception of biological processes. The influence of habitat colouration on proximate biological processes varies depending on the life history of an organism, and the nature of the inter- or intra-specific interaction being considered^4,28^. Within reef fish, juvenile survivorship shapes populations due to strong selective pressures^47,48^. Across a range of taxonomically diverse reef fishes, approximately 55% of juveniles are consumed within two days of reef settlement^47^. Due to their disproportionally high contribution to larval recruitment, cryptobenthic families, including Gobiidae, Apogonidae, and Syngnathidae, experience elevated juvenile mortality levels and comprise nearly 60% of consumed reef fish biomass^48^. Furthermore, at minimum, 126 fish species are closely associated with live coral habitats as juveniles prior to undergoing an ontogenetic shift at maturity^49^. The present study's observation that recruitment success declines as reef colour homogenizes, and the established importance of juvenile fish-coral associations, suggests the post-settlement environment, characterized primarily by coral richness, structure, and colouration, strongly influences fish families' association with coral richness and hue diversity^47,49,50^. This evidence contrasts the 'Lottery hypothesis' proposed by Sale^51^ and revisited by others^52^, which postulates that reef fishes' diversity within a trophic guild is primarily a consequence of competition for space and who gets there first. Consequently, the variability in morphology and hue among species within a guild is secondary or not relevant in an ecological or evolutionary context. Conversely, our results demonstrate a striking covariation among taxonomic diversity of fishes, corals and hue, in substantial contrast to a lottery mechanism as it suggests predictability and functionality of the unique attributes of each fish species on the reef.

The observed exceptionally high correlations between fish species richness and coral hue diversity indicate a functional contribution of colour to reef fishes' selective landscape. The Type-3 survivorship curves present in all reef fish populations are primarily structured by predation during early ontogeny^47,48,53^. Despite the five decades of studies of coral reef fishes^24^, there remains minimal empirical data on the multiple spectral backgrounds against which predators see different reef species ontogenetic size classes. This basic spatial geometry of predator–prey interactions can be fundamental to any interpretation of adaptation^8,9,54^. For example, colour diversity in intertidal gastropods indicates that the microhabitat of early ontogenetic stages comprises the major selective landscape influencing the diversity and fitness of phenotypes^4,55^. As such, the unique hue for each additional coral species may facilitate increased ecological opportunity for any of the variable colour phases during ontogeny. The combined effects of coral species diversity and hue diversity constitutes a plausible contributory mechanism for the origin and persistence of the taxonomic and spectral diversity in reef fishes^33^.

Fish perception of seascape colouration is fundamental to unravelling the influence that coral hues have on reef communities. At a minimum, our results represent an exceptionally conservative proxy for hue diversity perceived by reef fishes as they do not address the elevated hue sensitivity of most fishes relative to human colour perception^15,27^, the added effects of fluorescence present in multiple coral species^56^, and the contribution of additional brightly coloured taxa including encrusting and epiphytic algae, sponges, soft corals and crinoids. Reef fish exhibit di- and tri-chromacy, with evidence that a species’ photoreceptor spectral sensitivity is linked evolutionarily to the spectra it encounters^8,14,15^. This indicates that the hue diversity of corals, compounded by that of other benthic taxa, has been a significant driver in the evolution of reef fish photoreceptor spectral sensitivity. The ability of reef fish to alter their location, orientation, colouration, or pattern in relation to inter- and intra-specific perception further suggests that numerous aspects of reef fish behaviour, physiology, and community composition are functionally linked to coral colouration^12,27,28,57^. For example, various coral-associated species, including members of Gobiidae, Blennidae, and Monacanthidae, rapidly modify their luminance, hue, or saturation relative to background characteristics to avoid detection by visual predators^58–60^. Moreover, the conspicuous colouration observed in numerous reef fishes serves as a defensive strategy at close distances, while simultaneously blurring into the background when viewed from a distance^12,61^. Therefore, the observed exponential relationship between hue diversity and fish richness can be attributed to the myriad of biological processes facilitated by background colouration. Consequently, the visual interactions of reef fish are a function of the viewer’s visual acuity, the subject’s colouration and pattern, and the interplay between coral structure, colour, and the subject’s orientation relative to the background^12,14,27^. As ecological processes occur over a range of spatial scales, the influence of background coral colouration on the detection of fish colour and pattern represents a critical aspect of coral reef dynamics^12,14,27^, which supports taxonomically diverse fish communities, and on an evolutionary time scale promotes reef fish diversification.

The multitude of stressors that compromise the integrity of corals elicit local and global declines in hue diversity^62–64^. These devastating ecological events are natural experiments demonstrating coral hue’s contribution to reef ecosystems, particularly because coral colour loss commonly occurs prior to, or in the absence of, structural degradation^65–67^. Our evaluation of reef colour homogenization illustrates that bleaching creates a distinct monochromatic seascape, causing major reductions in fish richness, abundance, and in particular, recruitment success. A consequence of this is the recognition that coral hue within intact reefs promotes diverse fish communities, increases fish family-specific abundances, and raises recruit’s survivorship independent of coral species richness and structural complexity. Therefore, potential declines in fish communities will be highly influenced by a reef’s pre-bleaching coral richness and hue diversity, and the reef fish families present. The prominent rates of successful predation on bleached coral reefs and the strong association that numerous fish families have with coral colouration suggest that fish communities will be unlikely to recover if reefs remain devoid of colour^49,50^. Furthermore, if anthropogenic or natural stressors extirpated coral species and their associated hues, the extent to which fish communities can recover will be reduced accordingly, thereby threatening one of the highest concentrations of vertebrate diversity observed globally^25,32,68^.

Coral richness and colouration are among the most striking aspects of reef ecosystems. Despite this, the contribution of reef colouration in supporting diverse fish communities is frequently unrecognized. This omission is particularly evident when considering equivalent terrestrial ecosystems where substrate and background hue have featured prominently in ecological opportunities for invertebrate and vertebrate taxa^8,9^, and by association, verified the significance of species interactions with habitat colouration. The importance of coral reef colouration is becoming more apparent as the frequency and severity of reef disturbance events intensify, and the ecological ramifications of coral colour homogenization become increasingly evident^62–64^. This evaluation represents the first global and regional analysis quantifying the influence of coral richness and hue on reef fish communities. Examining either of these covariates independently would constitute a novel analysis, but when considered in combination, the established but unsubstantiated ecological importance of coral species diversity is coupled with each coral's spectral diversity. The evaluation revealed that the richness of coral reef fishes observed globally is sustained by regional coral species richness and functionally coupled to coral reefs hues' diversity. Whereby, each additional coral species contributes ecological opportunities to the surrounding seascape, which collectively provide the background heterogeneity required to support a diverse array of reef taxa. A pattern replicated across the diversity of coral reef fish and within 25 common fish families. With coral richness and hue functioning in combination to support diverse reef fish assemblages, we have described an aspect of coral reefs that is fundamental to a myriad of biological processes, that persists within and among fish families, varies according to the dynamic nature of reef ecosystems.

## Materials and methods

### Coral and fish richness

Coral and reef fish species data were compiled for 74 global ecoregions to evaluate the relationship between fish species richness, coral species richness, and coral colouration (Fig. 1). All available coral and fish species data were retrieved from Corals of the World^38^ and FishBase^26^, respectively. Ecoregions were constructed by adapting Corals of the World's 150 ecoregions and FishBase's survey locations to determine regions where both coral and fish surveys occurred^26,32,38^. The assessment of coral richness considered the 784 scleractinian coral species that occurred within the 74 ecoregions. Two examinations of reef fish richness occurred. Firstly, we evaluated reef fish intra-family species richness for the 25 most common families within the same ecoregions, which encompassed 3250 fish species. Secondly, we analyzed the distribution of 4465 fish species from 177 families within the ecoregions. Each examination of reef fish richness was combined with the 784 scleractinian corals’ distribution to generate reef fish and coral richness estimates within each of the ecoregions^40^ (Table S1 in Supporting Information). All data analyses were conducted in RStudio version 3.6.1^41^. Data visualizations were generated using the ‘ggplot2’, ‘colordistance’, ‘plot3D’ and ‘lattice’ packages^39,41,69–71^. All data and R code are available^40^.

### Coral image acquisition

Coral images were collected from the Corals of the World repository. This archive is the only publicly available coral image repository that includes at least one image of each scleractinian coral species that has been validated and quality controlled by experts in coral taxonomy^32,38^. For each of the 784 scleractinian coral species, the best quality photo was selected and downloaded as a JPEG image. Image acquisition targeted photos that displayed each species' typical appearance during the daytime, with consistent and appropriate lighting. Images with polyps fully or partially retracted were preferentially chosen, unless the species was known to open its polys diurnally. Images depicting rare colour morphs, irregular growth forms, or with distorted colouration were avoided. Each photo was cropped using ImageJ software to obtain an image containing only the focal coral species^72^. Image cropping removed the image background, other coral species, and any colour distortions. Minor shadows created by the focal coral were not removed, as they reflect the increased colour variance of structurally complex coral. All images were selected and processed by the same individual, with image cropping occurring in a random, non-taxonomically hierarchical order.

### Coral image colouration

Coral colour classification and categorization were conducted by integrating the Level 3 Inter-Society Colour Council and the National Bureau of Standard (ISCC-NBS) system into the recently developed ‘colordistance’ package (Fig S1)^39,73^. The selection of this colour classification and categorization was validated relative to alternative colouration systems (Supplemental Text, Fig S1-S6). The Level 3 ISCC-NBS system calculates centroids based on Munsell colour space to produce 267 distinct colour categories. The ISCC-NBS centroids can also be considered in terms of sRGB colour space, as all sRGB denominations can be allotted into 260 of the ISCC-NBS categories^74^. The ‘colordistance’ package quantitatively derives colour trait data from images and is capable of comparing the similarity of different colour palettes, which allows for coral images’ colour palettes to be quantified, categorized according to the Level 3 ISCC-NBS colour system, and analyzed accordingly.

The colour diversity of each of the 784 coral images was determined independently. All aspects of image colour acquisition and subsequent statistical analyses were performed using R statistical software version 3.6.1^41^. Each image's pixels were considered as three-dimensional coordinates in colour space to create a discrete multidimensional colour histogram (Figs. 1, S1). The maximum number of histogram bins was set a priori at 27, which corresponds to the number of regions in colour space that each of the standard red–green–blue (sRGB) channels were divided into (3 colour regions, 3 channels, 3^3^ = 27 bins; Figs. 1, S1). The number of histogram bins occupied, the bin’s sRGB colour composition, and the number of pixels allotted to each bin was a function of the image’s colour diversity and the proportion of each colour present. To reduce the risk that abnormalities within the image, specifically small portions of discoloured pixels, were integrated into the analysis, bins that obtained less than 1% of the pixels were excluded. Each histogram bin was converted from its sRGB coordinates to ISCC-NBS, using hexadecimal colour codes as an intermediate conversion step. This resulted in coral colour richness illustrated by the number of visually distinct colour stimuli present within each image. The colour diversity of each ecoregion was determined by summarizing the number of unique colours present, given its coral composition (Fig S3). Across all corals, 199 of the possible 267 ISCC-NBS colours were observed, with the number of unique colours within each coral family, and detected across ecoregions, varying considerably (Figs. S3–S5).

### Coral, fish, and colour analyses

The influence of coral richness and hue diversity on fish species assemblages was examined within each of the 25 reef-associated fish families. The primary analysis examined this relationship among the four oceanic regions, and a subsequent examination considered this relationship within the oceanic regions. Multiple linear regression models evaluated the influence of coral richness and hue diversity on reef fish species richness within each family. Coral richness was incorporated as a second-order polynomial to account for the relationship between coral, hue and fish richness being non-linear in multiple instances. Specifically, the addition of a polynomial term accounted for occurrences when increasing hue diversity was highly correlated with fish richness, despite minimal coral richness (i.e. a few vividly coloured corals supporting diverse fish communities). Additionally, the influence of coral richness and hue diversity on the 25 reef-associated fish families was also considered independently.

The correlation between coral species richness, associated hue diversity, and reef fish assemblages within each ecoregion was subsequently evaluated using a combination of linear and non-linear models. The initial analyses considered the distribution of 4465 fish species from 177 families, 784 coral species, and coral colouration, across the 74 ecoregions (Table S1). A linear model assessed the correlation between coral and fish richness. To account for reef colour saturation, a logistic regression model quantified the relationship between coral richness and hue diversity. A quadratic (second-order) polynomial model assessed the relationship between fish richness and unique hue diversity. A multiple regression quantified the influence of hue diversity, and coral richness as a quadratic (second-order) polynomial term, on reef fish richness. Akaike's Information Criteria evaluated coral richness and hue diversity, when modelled separately and in combination, as predictors of fish species richness (Table S2). Separate multiple regressions quantified the influence of hue diversity, and coral richness as a quadratic (second-order) polynomial term, on the reef fish richness observed within each of the four oceanic regions (Fig S7).

### Reef colour analysis

To evaluate hue diversity across coral reef seascapes and quantify coral colour loss, reef images taken by the Ocean Agency during the XL Catlin Seaview Survey were collected from Coral Reef Image Bank. Survey images of shallow water healthy reefs under bright ambient lighting in American Samoa (Fogama), Australia (Lizard Island), Indonesia (Manado), Taiwan (Donghi harbor), Turks and Caicos (Providenciales), and a severely bleached reef surveyed in American Samoa were selected for analysis^42^. The XL Catlin Seaview Survey images were chosen to increase consistency across the photos. Image selection emphasized wide-angle survey images that captured a diversity of coral reef taxa, including stony and soft corals, sponges, other invertebrates, and small reef-dwelling fish. These images were cropped to remove the water column.

A distance matrix compared hue similarity between the Catlin Seaview Survey images. A discrete multidimensional colour histogram considered each survey image in sRGB colour space. Sixty-four histogram bins (4 colour regions, 3 sRGB channels, 6^3^ = 64 bins) were used to account for the increased hue diversity relative to the single coral images previously analyzed. Each bin’s associated sRGB colour space denomination, the proportion of pixels within each bin, and the number of bins occupied across the histogram, were functions of each reef’s hue diversity. The colour distance matrix considered the pairwise distances between survey images using earth mover’s distance, a technique that compares histograms using transportation costs (i.e. the effort required to make one community resemble another). The pairwise distances between reefs were visualized by plotting a heatmap of the symmetrical distance matrix. The resulting heatmap illustrated the hue similarity between the reefs considered, with the plotted branch lengths being proportional to the earth mover distances.

An examination of published literature was integrated into this analysis to determine the ecological consequences of coral colour loss (Table S3 in Supporting Information, Appendix 1 Data Sources). Data were extracted and summarized from studies that evaluated reef fish responses to bleaching events that induced colour loss but maintained coral richness and structure (Supplemental Text). The criteria that studies had to be explicit about coral richness and structure being preserved limited the potentially relevant literature on the topic considerably. Data from 133 comparisons were extracted and summarized data from eight studies (Table S3). The ‘Metafor’ package was used to calculate the standardized mean difference (Hedge’s d) and the corresponding variance of each comparison^41,75^. Effectively, this determined the overall effect of colour loss on fish richness, abundance, recruitment, or fish family-specific abundances.

## Supplementary Information

Supplementary Information.

## Data Availability

The authors declare that the coral, fish and hue data supporting the findings of this study are available within the paper and provided as raw data files. The coral images are available online through the Corals of The World website www.coralsoftheworld.org or from the corresponding author upon request. The R code supporting the findings of this study are provided. All data, images, and code are also available through Figshare with the identifier ‘10.6084/m9.figshare.12317591’ and will be published upon manuscript acceptance.
