# Supplementary material for: Regional heterogeneity in coral species richness and hue reveals novel global predictors of reef fish intra-family diversity
Source: Sci Rep. 2021 Sep 14;11:18275. doi: 10.1038/s41598-021-97862-8 (PMC8440613; doi:10.1038/s41598-021-97862-8)
Supplement: Supplementary file 1 — Supplementary Information. [file 41598_2021_97862_MOESM1_ESM.docx]

**Supporting Information For**

**Title:** Regional Heterogeneity in Coral Species Richness and Hue Reveals Novel Global Predictors of Reef Fish Intra-Family Diversity

**Authors:** Kieran D. Cox^1,2*^**,** Mackenzie B. Woods^1^, Thomas E. Reimchen^1^*

**Affiliations:**

^1^ Department of Biology, University of Victoria, Victoria, British Columbia, Canada, V8P 5C2

^2^ Hakai Institute, Heriot Bay, British Columbia, Canada, V0P 1H0

*Correspondence to Thomas E. Reimchen and Kieran D. Cox. Email: reimchen@uvic.ca and kcox@uvic.ca. Mail: Department of Biology, University of Victoria, Cunningham 202, 3800 Finnerty Road, Victoria, BC V8P 5C2, Canada. Phone: 250-721-7101 Fax: 250-721-7120

**This PDF file includes:**

Supporting Text: Methods and Materials

Appendix 1 Data Sources

Figures S1-S10

Supporting Tables: S1-S3

Supporting References: 29

**Other supplementary materials for this manuscript include the following:**

Datasets S1 to S14

R Code S1

**Supplemental Methods and Materials**

**Image Colouration Method Validation**

To validate the coral colour classification and catalogue systems, we empirically evaluated potential alternatives for each stage of the methodology. Within the colourdistance package, the number of histogram bins derived from each image was varied from 1-125 bins (via 1,2,3,4 and 5 channels) to ensure the coral colour diversity was adequately captured (Fig. S2). This step identified 27 histogram bins, corresponding to 3 colour regions within each of the sRGB channels, as suitable for adequately capturing colour diversity. Under this condition, across all corals, 199 of the possible 267 ISCC-NBS colours were observed (Fig. S4). The influence of utilizing different colour catalogues to convert image histogram bins sRGB denominations into distinct colours system was also explored. Empirically, the implications of considering coral colouration in terms of hexadecimal colour codes, Level 3 ISCC-NBS, and a modified hexadecimal colour code system that reduced the number of detectable colours to 1,331, were examined (Fig. S2). The use of ISCC-NBS Level 1 and 2 Categories, HSV (Hue, Saturation, Value), and CMYK (Cyan-Magenta-Yellow-black), were considered but not empirically quantified given evident limitations in these colour systems. CIELab space was considered as it converts RGB pixels into a colour space that is representative of the degree that human vision can perceive differences between colours. As CIELab colour analysis requires information on the photographic lighting condition (e.g. incandescent bulb, daylight, indirect sunlight), it was excluded as meeting this condition was impossible for the 784 coral images analyzed.

The image selection process was evaluated using a multiple method approach focused on addressing concerns surrounding coral phenotypic variation and coral intraspecific variation (within the same phenotype). The implications of coral phenotypic variation on colour diversity was examined using three images of *Acanthastrea ishigakiensis* individuals that exhibited distinct colour morphs (Fig. S6). Coral intraspecific variation was assessed in a similar manner, with different *Acropora verweyi* individuals of the same colour morph being analyzed (Fig. S6). The *A. ishigakiensis* and *A. verweyi* images were selected and cropped in accordingly. The colour diversity within each of the *A. ishigakiensis* and *A. verweyi* images was determined by following the same procedure as the 784 images previously analyzed.

The influence of image format on observed colour diversity was considered extensively. However, as the ‘colourdistance’ analysis utilized can only be conducted on JPEG or PNG images, an analysis comparing RAW, JPEG, PNG, TIFF or other image formats could not be conducted ^1^. Furthermore, RAW images would be preferential to JPEG image ^2^ due to information loss during compression, but RAW images do not exist for even a fraction of the 784 corals analyzed. As JPEG images are readily utilized for the study of animal colouration, patterns, colour theory, and visually mediated ecological interactions ^3–10^, it was concluded that the approach taken was appropriate. Had a format that imbeds more colour information been utilized, heightened colour diversity may have been detected and as such the derived colour diversity may be an underestimate.

**Literature Analysis**

We analyzed published literature to determine the ecological consequences of coral colour loss. We extracted and summarized data from studies that evaluated reef fish responses to bleaching events that induced colour loss but maintained coral richness and structure (Table S3, Appendix 1 Data Sources). The criteria that studies had to be explicit about coral richness and structure being preserved limited the potentially relevant literature on the topic considerably. Commonly, incorporated studies involved fish surveys occurring before and immediately after a bleaching event, or during a bleaching event and then after the corals had recovered. Among the studies considered, the comparison of bleached and unbleached reefs occurred both spatially and temporally. The reef fish metrics (i.e. response variables) surveyed included fish richness, abundance, and the success/persistence of fish recruitment events. In instances where studies reported fish family-specific abundances, the families surveyed were documented. This condition occurred in a limited number of cases, as surveys of fish abundances commonly assessed entire communities (Table S3).

We extracted and summarized data from eight studies. Within each study, the control and experimental means, associated sample sizes and variances (standard error or standard deviation) were extracted from each comparison of the response variables (e.g. fish richness, abundance, recruitment or fish family-specific abundance) under bleached and unbleached conditions. Consequently, data from 133 comparisons were extracted from the eight studies. The ‘Metafor’ package was used to calculate the standardized mean difference (Hedge’s d) and the corresponding variance of each comparison^11,12^. Each comparisons Hedge’s d and corresponding variance were aggregated according to the response variable evaluated and averaged at the study level. Therefore, when studies examined multiple response variables, they were considered independently, and the comparisons within each response variable were aggregated accordingly. This approach was warranted as the response variables effect sizes were never pooled during the analysis. Within each response variable, the ‘MAd’ package calculated the aggregate dependent effect sizes and variances by averaging the studies Hedge’s d and variance ^13^. This process combined all within-study effects prior to evaluating among study effects. Effectively, this determined the overall effect of colour loss on fish richness, abundance, recruitment, or fish family-specific abundances.

**Limitations**

Our aim was not to characterize and quantify the specific colours found on coral reefs; limitations surrounding an ecologically relevant and globally accepted colour classification system make this impractical. Instead, we concentrated our efforts on integrating an established colour classification system and comprehensive colour catalogue into coral reef ecology ^1,14^. This resulted in coral colour assessments that are informative and contextual within the framework of our analysis.

Our assessment of coral richness and hue’s influence on reef fish communities globally represents a considerable development in our understanding of how to integrate the contribution of background colourations into the study of ecological communities. This investigation was only achievable due to innovations in the quantitative colour profiling of digital images, and the enduring efforts of those developing and maintaining global marine diversity databases^1,15,16^. However, many challenges still exist when analyzing the hue diversity of natural systems, especially when using rapidly evolving, yet still limited, technologies. This is particularly true of digitizing images into biologically relevant colour diversity. Specifically, the sRGB format of digital images is modeled after human colour vision and a poor proxy for non-human perception ^2,17,18^. Despite lacking a biologically relevant colour classification system, image calibration and colour space conversion can still provide meaningful biological insights ^1,6,8,10^. Many other challenges, using JPEG images instead of preferential RAW images, for example, are currently unavoidable given photo archive limitations. Despite these limitations, the analyzing images of coral, including determining characteristics of coral colouration, has been an invaluable aspect of coral reef ecology for decades ^19–24^. Furthermore, natural marine conditions exhibit considerable variation in visual conditions (e.g. light, depth, clarity) and coral colour, and contribute additional colouration via other benthic substrates and invertebrates (e.g. sponges, soft corals). This plethora of colour diversity was not considered within this analysis and implies that our current interpterion of colour’s contribution to ecological communities is an underestimate, as natural systems and their colouration are much more dynamic and diverse.

**Appendix 1 Data Sources:**

Data sources utilized in the literature analysis to construct Figure 5.

Bonin, M., Munday, P., McCormick, M., Srinivasan, M. & Jones, G. (2009) Coral-dwelling fishes resistant to bleaching but not to mortality of host corals. *Marine Ecology Progress Series*, **394**, 215–222.

Booth, D. & Beretta, G. (2002) Changes in a fish assemblage after a coral bleaching event. *Marine Ecology Progress Series*, **245**, 205–212.

Coker, D.J., Pratchett, M.S. & Munday, P.L. (2009) Coral bleaching and habitat degradation increase susceptibility to predation for coral-dwelling fishes. *Behavioral Ecology*, **20**, 1204–1210.

Coker, D.J., Pratchett, M.S. & Munday, P.L. (2012) Influence of coral bleaching, coral mortality and conspecific aggression on movement and distribution of coral-dwelling fish. *Journal of Experimental Marine Biology and Ecology*, **414–415**, 62–68.

Feary, D., Almany, G., Jones, G. & McCormick, M. (2007a) Coral degradation and the structure of tropical reef fish communities. *Marine Ecology Progress Series*, **333**, 243–248.

Feary, D.A., Almany, G.R., McCormick, M.I. & Jones, G.P. (2007b) Habitat choice, recruitment and the response of coral reef fishes to coral degradation. *Oecologia*, **153**, 727–737.

McCormick, M.I., Moore, J.A.Y. & Munday, P.L. (2010) Influence of habitat degradation on fish replenishment. *Coral Reefs*, **29**, 537–546.

Yahya, S.A.S., Gullström, M., Öhman, M.C., Jiddawi, N.S., Andersson, M.H., Mgaya, Y.D. & Lindahl, U. (2011) Coral bleaching and habitat effects on colonisation of reef fish assemblages: An experimental study. *Estuarine, Coastal and Shelf Science*, **94**, 16–23.

**Figures**


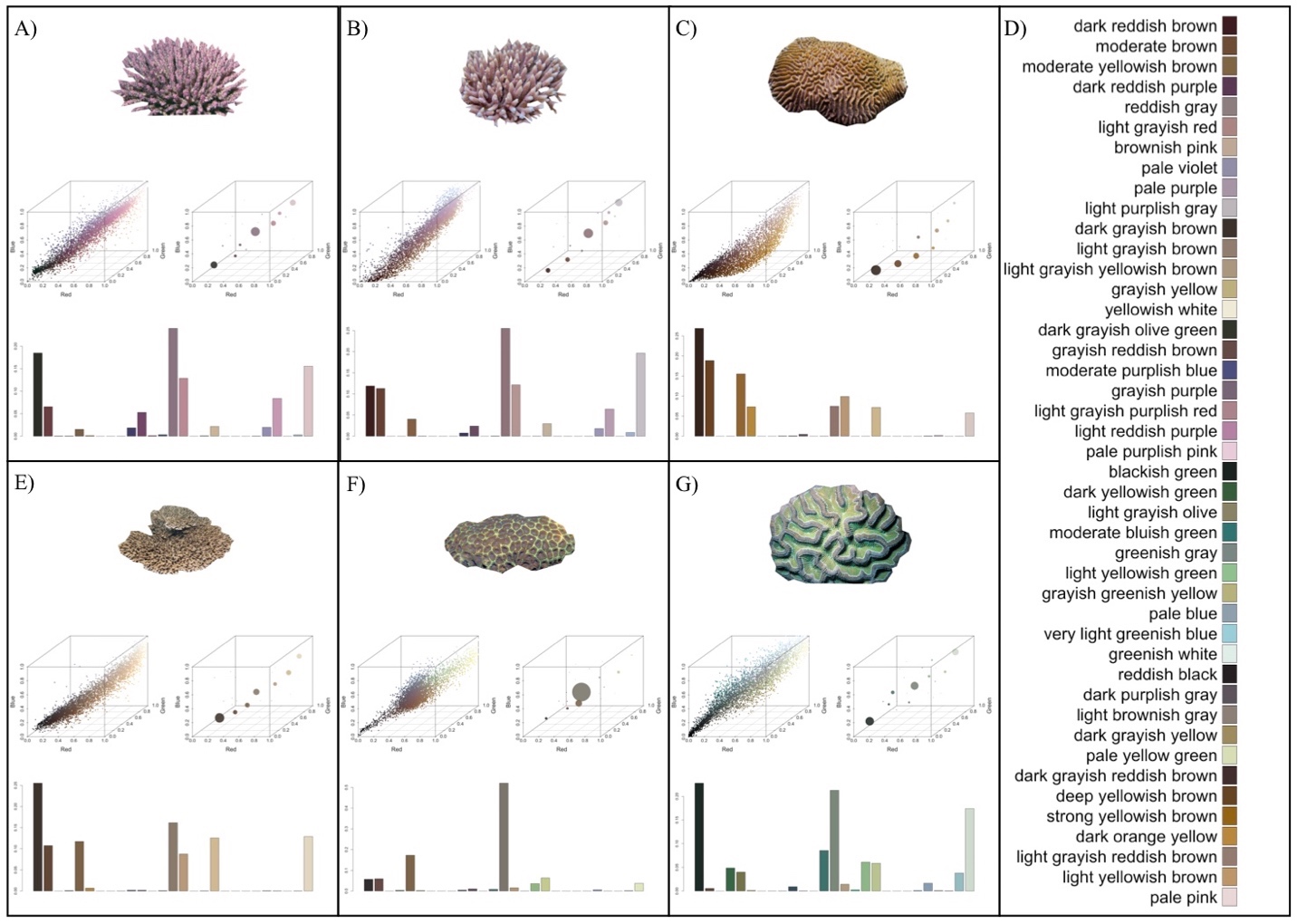


**Fig. S1. (A-C, E-G)** Examples of coral hue quantification. Illustrates the cropped coral images, associated hue profiles in RGB colour space, and the resulting colour histogram bins. **A)**Acropora subulata**B)**Acropora millepora**C)**Platygyra acuta**D)**Level 3 ISCC-NBS colours detected across the example corals**E)**Acropora solitaryensis**F)**Favites bestae**G)**Colpophyllia natans.

**
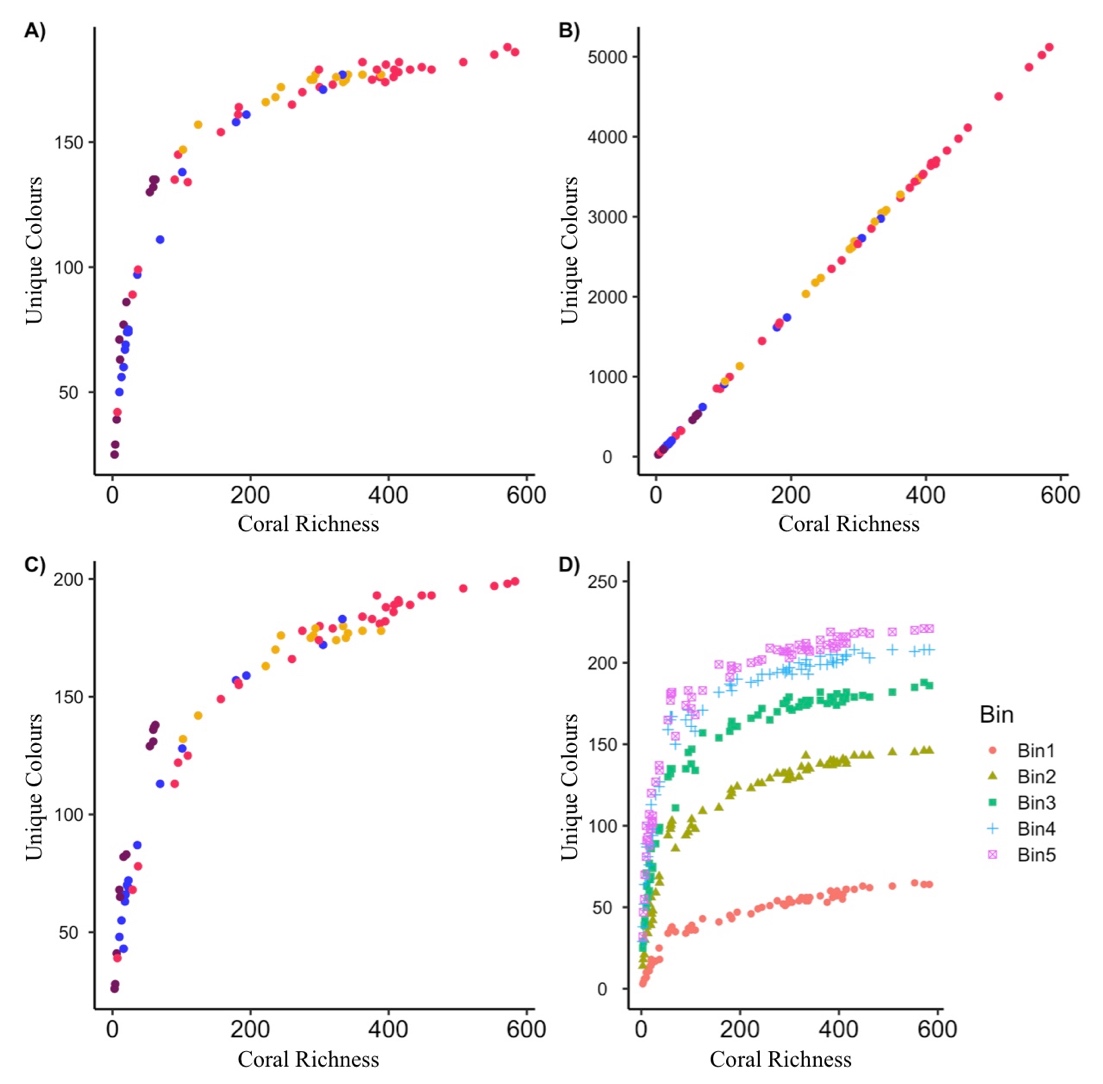
**

**Fig. S2.** Colours diversity detected when analyzing 784 scleractinian corals using three different colour classification systems. Each point denotes an ecoregion, and oceanic regions are represented by distinct colours: **A-C)** Atlantic—yellow, Northern Indian Ocean—blue, Central and East Pacific—purple, Western Pacific—red. **A)** Level 3 ISCC-NBS colour system (267 possible colours) **B)**Hexadecimal Colour Codes**(**16,777,216 possible colours). **C)**A modified hexadecimal colour code system (1,331 possible colours). **D)** Hue diversity detected when analyzing 784 scleractinian corals while varying the number of bins from 1–5, resulting in 1, 8, 27, 64, and 125 histogram bins, respectively. Each coral image pixels are allotted in a discrete multidimensional colour histogram with the number of potential bins set a priori.


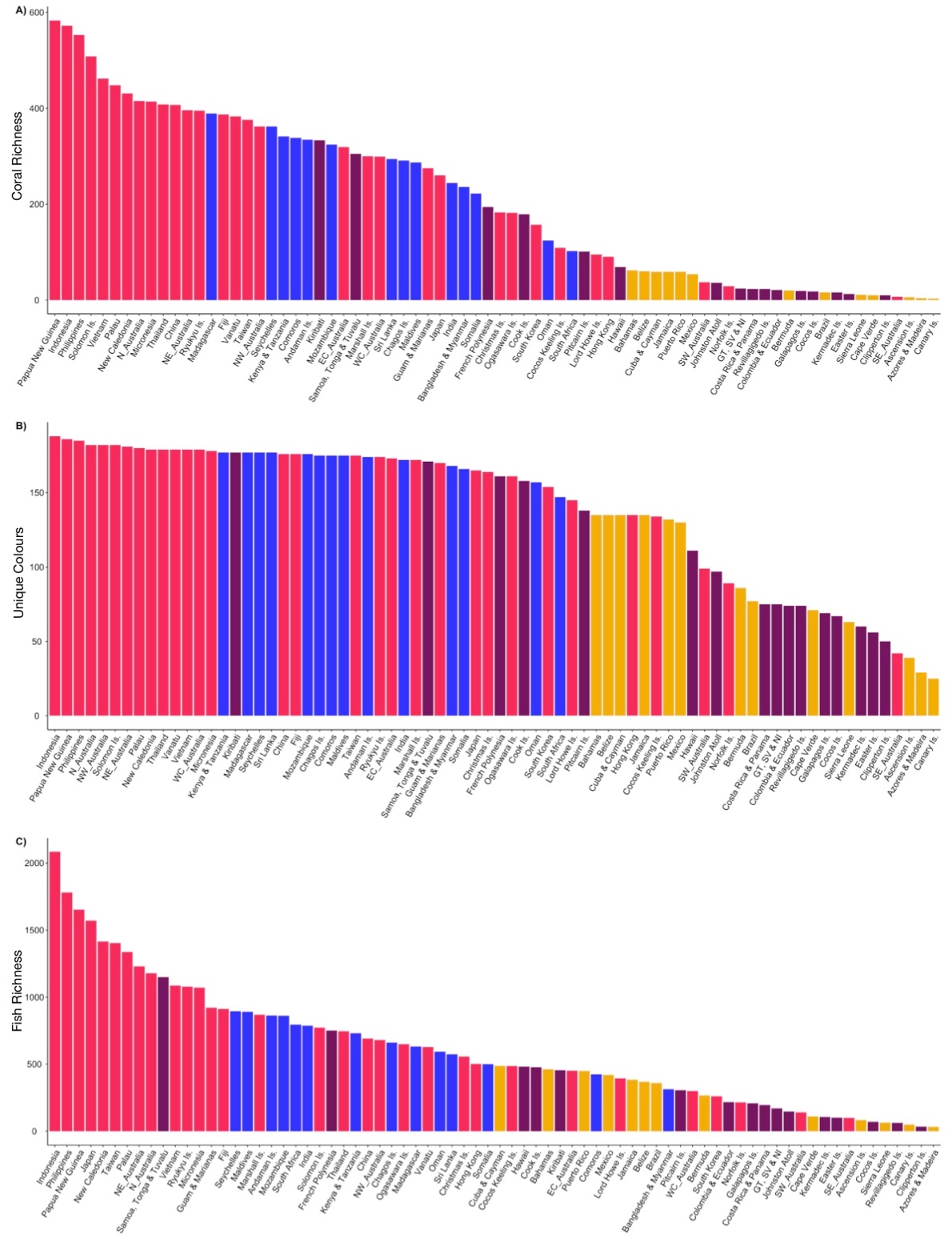


**Fig. S3. A)** Coral richness within 74 global ecoregions **B)**Colour diversity within 74 global ecoregions**C)**Fish richness within 74 global ecoregions**A-C)**Each bar denotes an ecoregion. Oceanic regions are represented by distinct colours: Atlantic—yellow, Northern Indian Ocean—blue, Central and East Pacific—purple, Western Pacific—red.


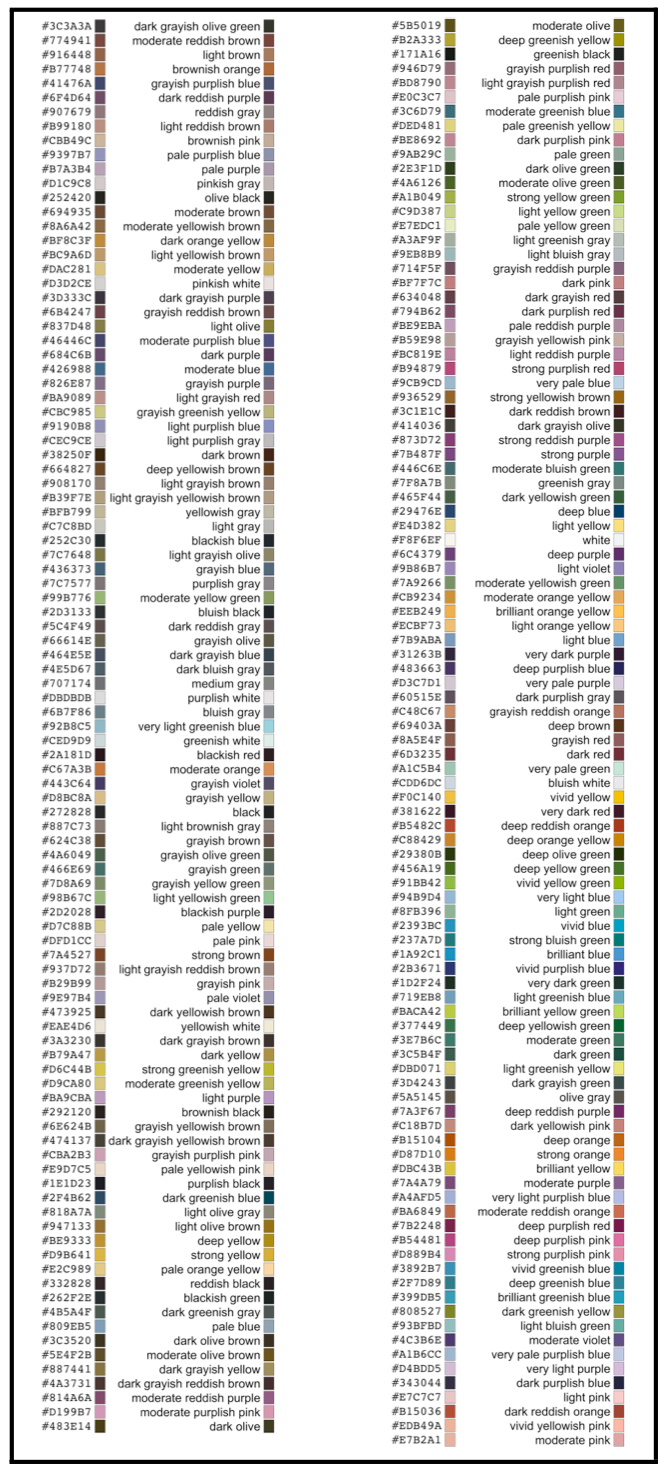


**Fig. S4.** Red-green-blue colour diversity detected when analyzing 784 scleractinian corals. Hexadecimal colour codes and equivalent Level 3 ISCC-NBS colours depicted. 199 unique colours detected across all corals.


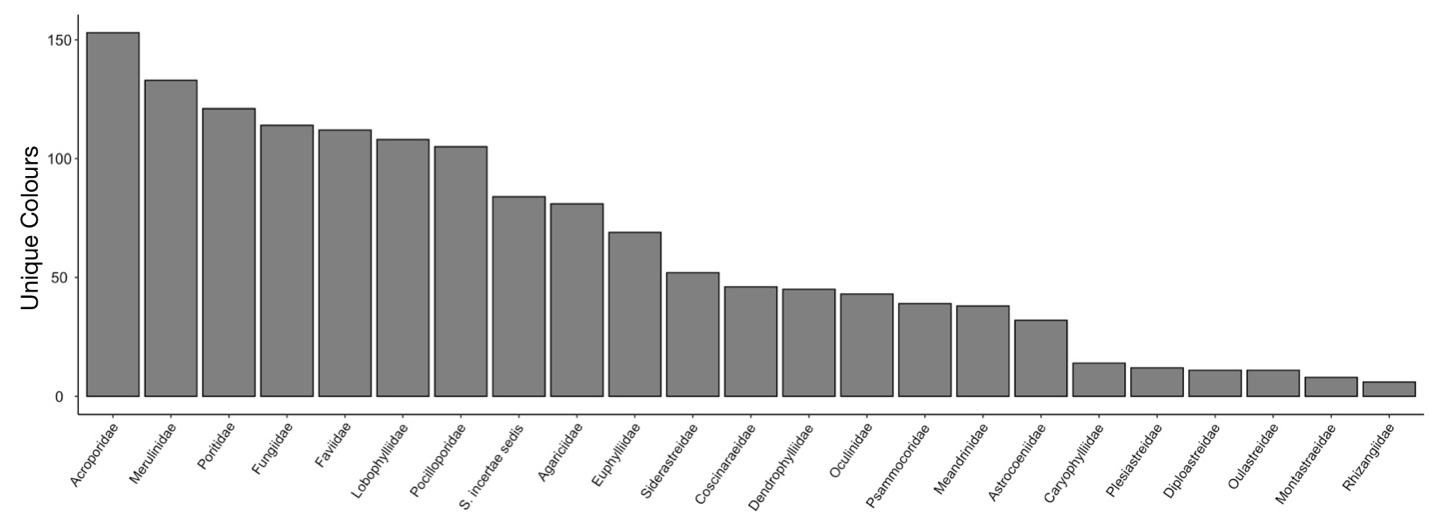


**Fig. S5.** Level 3 ISCC-NBS colours detected within 23 coral families. Colour diversity was assessed within 784 scleractinian corals prior to being pooled according to coral family.


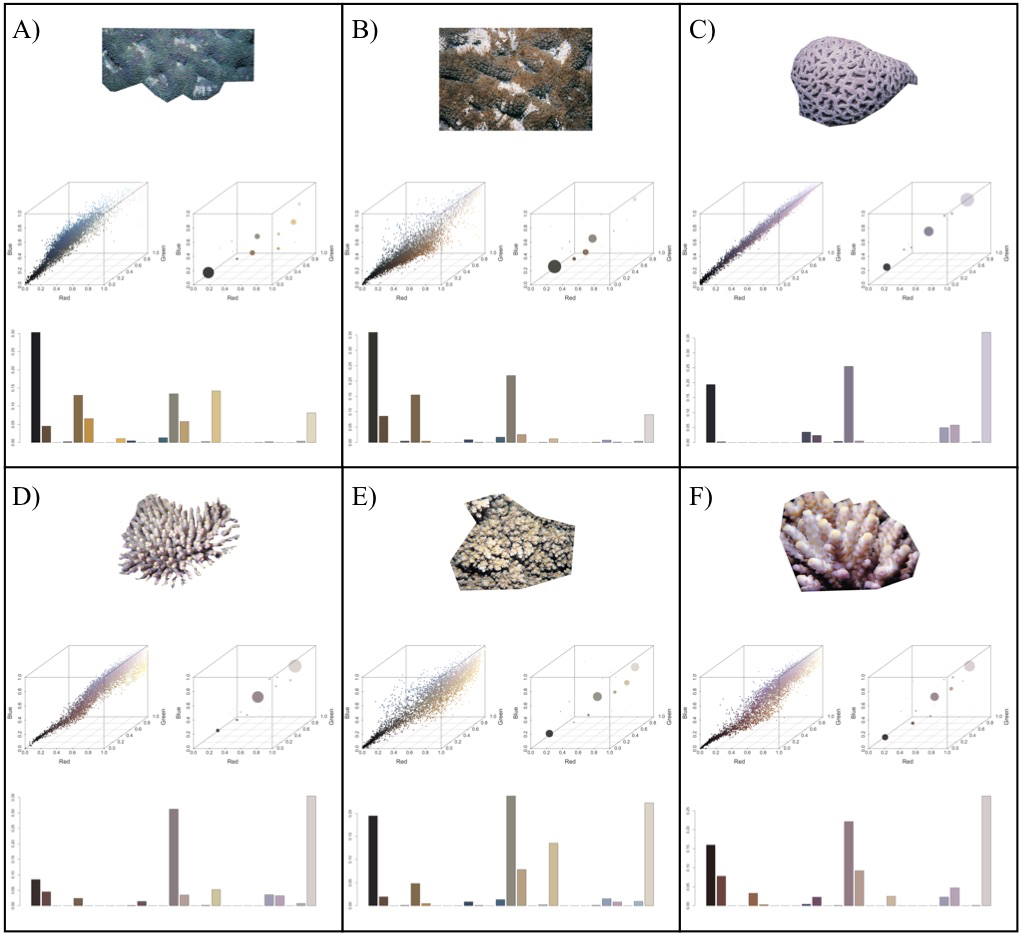


**Fig. S6. A-C)** Coral hue diversity quantification when analyzing three Acanthastrea ishigakiensis colour morphs that exhibit distinct phenotypic variability. Illustrates the cropped coral images, associated hue profiles in RGB colour space, and the resulting colour histogram bins (hue diversity). **D-F)** Coral hue diversity quantification when analyzing three Acropora verweyi specimens that exhibit intraspecific colour variability within the same colour morph. Illustrates the cropped coral images, associated hue profiles in RGB colour space, and the resulting colour histogram bins (hue diversity).


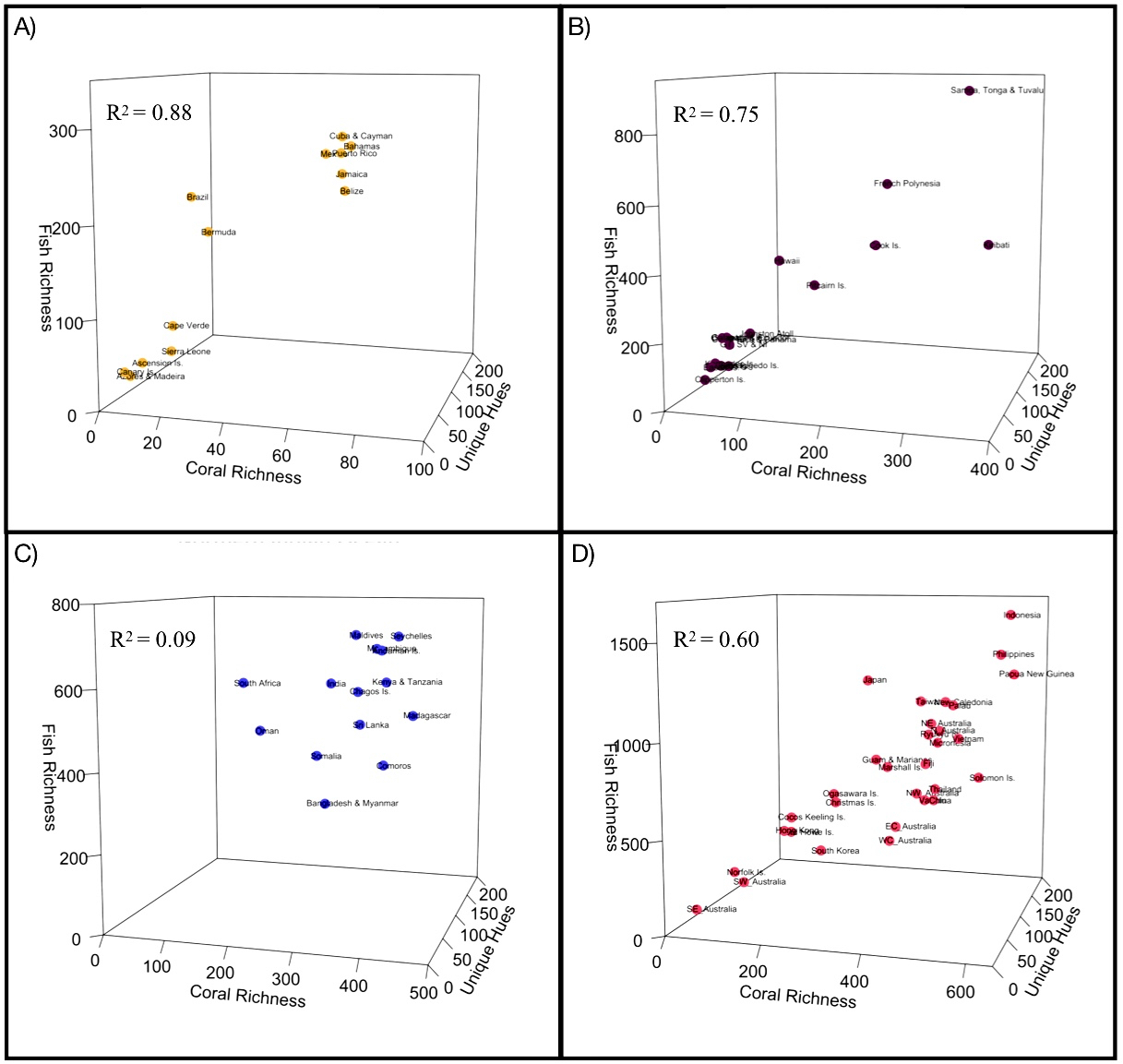


**Fig. S7.** The relationship between 784 coral species, fish species richness within 25 common reef families, and coral hue across 74 ecoregions. Each point denotes an ecoregion. Multiple regression evaluating the influence of hue diversity, and coral richness as a quadratic (second-order) polynomial term, on reef fish richness. Adjusted R-Squared values reported. **(A)** Atlantic **(B)** Central and East Pacific **(C)** Northern Indian Ocean **(D)** Western Pacific.

**
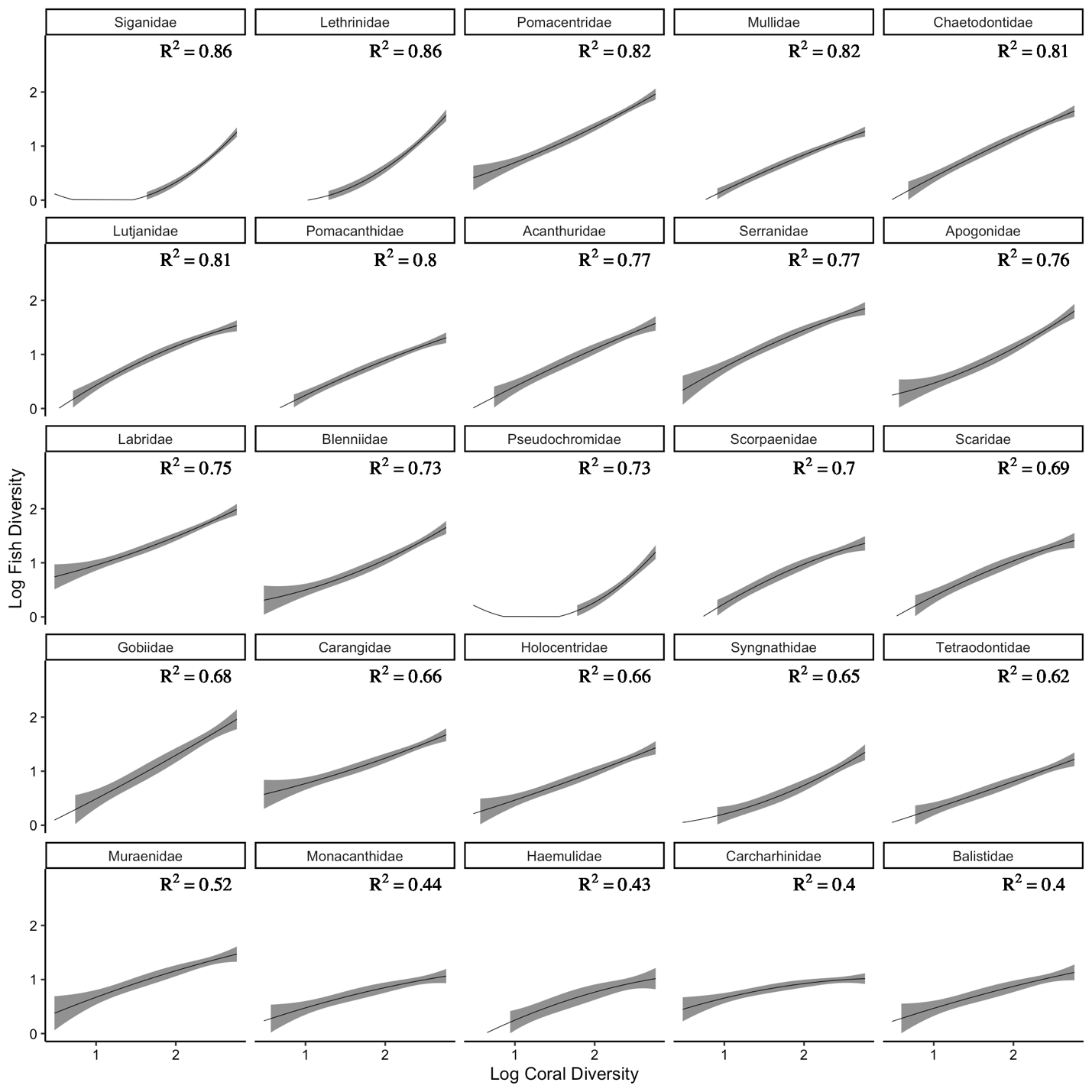
**

**Fig. S8. |** The relationship between fish intra-family richness, coral species richness, and coral hue diversity, within 25 common reef-associated fish families in each of the 74 ecoregions. Ecoregion points denoted in Figure 3 have been removed to better illustrate 95% confidence intervals, which are shown in grey. Fish families are ordered by r-squared value.


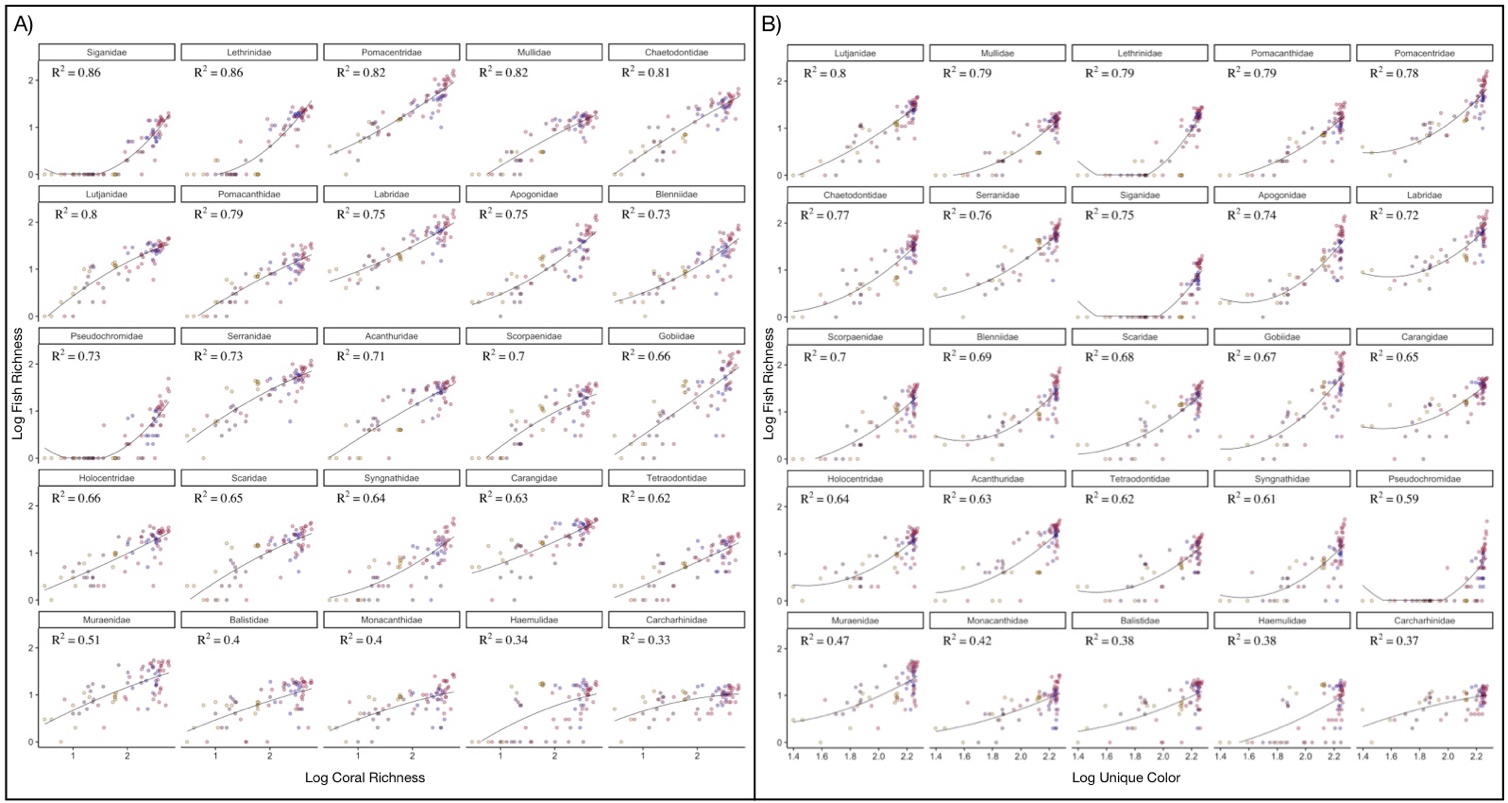


**Fig. S9. A)** The relationship between intra-family fish richness and coral richness within 25 common reef-associated fish families in each of the 74 ecoregions. **B)** The relationship between intra-family fish richness and coral hue diversity within 25 common reef-associated fish families in each of the 74 ecoregions. **A-B)** Each point denotes an ecoregion and oceanic regions are represented by distinct colours: Atlantic—yellow, Northern Indian Ocean—blue, Central and East Pacific—purple, Western Pacific—red.


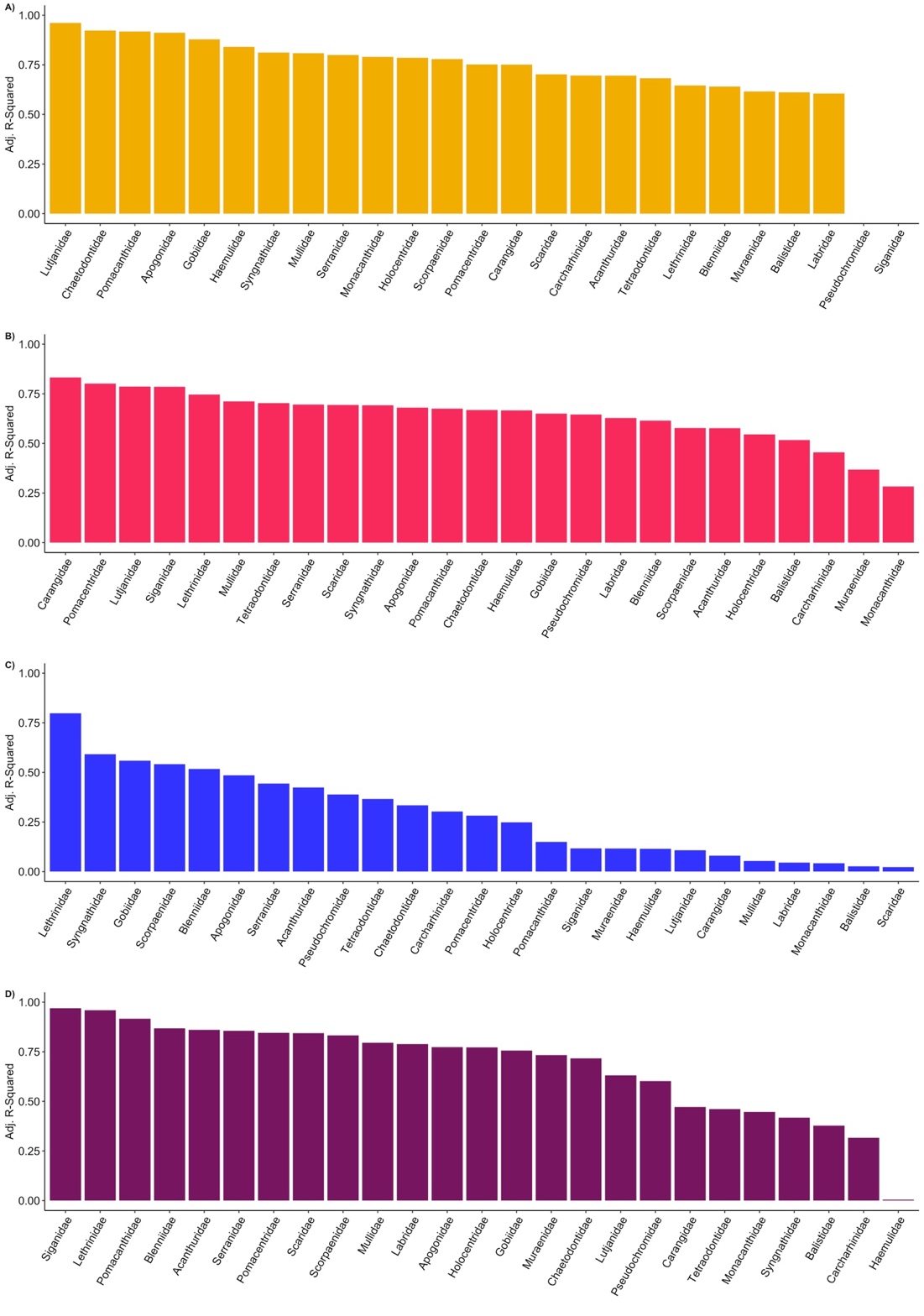


**Fig. S10.** The relationship between intra-family fish richness, coral species richness, and coral hue diversity, within each of the four oceanic regions. Fish richness, coral richness and hue were considered within 74 ecoregions. The 25-common reef-associated fish families are ordered by r-squared values. **(A)** Atlantic **(B)** Central and East Pacific **(C)** Northern Indian Ocean **(D)** Western Pacific.

**Tables**

**Table S1.** Coral richness, coral hue diversity, and fish richness across 74 ecoregions. Coral richness and coral hue diversity considered 784 scleractinian coral. Fish Richness denotes the distribution of 4,465 fish species from 117 fish families, whereas Fish Richness 25 denotes the distribution of 3,250 fish species from 25 of the most common reef-associated fish families.

| Oceanic Region | Ecoregion | Fish Richness | Fish Richness 25 | Hue Diversity | Coral Richness |
| --- | --- | --- | --- | --- | --- |
| Atlantic | Ascension Is. | 83 | 37 | 39 | 6 |
|  | Azores & Madeira | 32 | 26 | 29 | 4 |
|  | Bahamas | 461 | 269 | 135 | 62 |
|  | Belize | 368 | 216 | 135 | 60 |
|  | Bermuda | 266 | 174 | 86 | 20 |
|  | Brazil | 358 | 216 | 77 | 16 |
|  | Canary Is. | 49 | 32 | 25 | 3 |
|  | Cape Verde | 109 | 67 | 71 | 10 |
|  | Cuba & Cayman | 486 | 280 | 135 | 59 |
|  | Jamaica | 383 | 236 | 135 | 59 |
|  | Mexico | 419 | 260 | 130 | 54 |
|  | Puerto Rico | 448 | 261 | 132 | 59 |
|  | Sierra Leone | 63 | 41 | 63 | 11 |
| Central & East Pacific | Clipperton Is. | 34 | 30 | 50 | 10 |
|  | Cocos Is. | 69 | 55 | 67 | 18 |
|  | Colombia & Ecuador | 217 | 138 | 74 | 21 |
|  | Cook Is. | 476 | 385 | 158 | 179 |
|  | Costa Rica & Panama | 196 | 132 | 75 | 23 |
|  | Easter Is. | 100 | 63 | 56 | 13 |
|  | French Polynesia | 751 | 591 | 161 | 194 |
|  | Galapagos Is. | 208 | 142 | 69 | 19 |
|  | GT, SV & NI | 170 | 114 | 75 | 24 |
|  | Hawaii | 481 | 359 | 111 | 69 |
|  | Johnston Atoll | 146 | 128 | 97 | 36 |
|  | Kermadec Is. | 105 | 71 | 60 | 16 |
|  | Kiribati | 455 | 389 | 177 | 333 |
|  | Pitcairn Is. | 305 | 257 | 138 | 101 |
|  | Revillagigedo Is. | 61 | 46 | 74 | 23 |
|  | Samoa, Tonga & Tuvalu | 1149 | 900 | 171 | 305 |
| Northern Indian Ocean | Andaman Is. | 863 | 656 | 174 | 334 |
|  | Bangladesh & Myanmar | 312 | 217 | 168 | 236 |
|  | Chagos Is. | 661 | 538 | 175 | 291 |
|  | Comoros | 423 | 331 | 175 | 338 |
|  | India | 787 | 561 | 172 | 244 |
|  | Kenya & Tanzania | 730 | 566 | 177 | 341 |
|  | Madagascar | 632 | 474 | 177 | 389 |
|  | Maldives | 891 | 698 | 175 | 287 |
|  | Mozambique | 862 | 660 | 176 | 324 |
|  | Oman | 592 | 427 | 157 | 124 |
|  | Seychelles | 895 | 695 | 177 | 362 |
|  | Somalia | 499 | 356 | 166 | 222 |
|  | South Africa | 794 | 566 | 147 | 102 |
|  | Sri Lanka | 573 | 444 | 177 | 294 |
| Western Pacific | China | 692 | 485 | 176 | 407 |
|  | Christmas Is. | 556 | 460 | 164 | 183 |
|  | Cocos Keeling Is. | 485 | 406 | 134 | 109 |
|  | EC_Australia | 450 | 313 | 173 | 319 |
|  | Fiji | 912 | 703 | 176 | 387 |
|  | Guam & Marianas | 922 | 725 | 170 | 275 |
|  | Hong Kong | 501 | 320 | 135 | 90 |
|  | Indonesia | 2083 | 1594 | 188 | 572 |
|  | Japan | 1570 | 1206 | 165 | 260 |
|  | Lord Howe Is. | 393 | 297 | 145 | 95 |
|  | Marshall Is. | 869 | 680 | 172 | 300 |
|  | Micronesia | 1071 | 833 | 178 | 414 |
|  | N_Australia | 1178 | 900 | 182 | 415 |
|  | NE_Australia | 1229 | 942 | 181 | 396 |
|  | New Caledonia | 1414 | 1078 | 179 | 431 |
|  | Norfolk Is. | 215 | 155 | 89 | 29 |
|  | NW_Australia | 680 | 510 | 182 | 362 |
|  | Ogasawara Is. | 650 | 515 | 161 | 182 |
|  | Palau | 1336 | 1059 | 180 | 448 |
|  | Papua New Guinea | 1652 | 1247 | 186 | 583 |
|  | Philippines | 1780 | 1362 | 185 | 553 |
|  | Ryukyu Is. | 1078 | 884 | 174 | 395 |
|  | SE_Australia | 98 | 42 | 42 | 7 |
|  | Solomon Is. | 772 | 627 | 182 | 508 |
|  | South Korea | 260 | 175 | 154 | 157 |
|  | SW_Australia | 139 | 75 | 99 | 37 |
|  | Taiwan | 1403 | 1081 | 175 | 376 |
|  | Thailand | 746 | 549 | 179 | 408 |
|  | Vanatu | 628 | 477 | 179 | 383 |
|  | Vietnam | 1086 | 861 | 179 | 462 |
|  | WC_Australia | 299 | 211 | 179 | 299 |

**Table S2.** Model selection table based on corrected Akaike information criterion (AICc). K= the number of estimated parameters for each model. The Delta AIC= for the m^th^ candidate model is the difference between AICm and AICc, which indicates the support for each candidate. AICc Wt = Akaike weights, which indicate the level of support (i.e. evidence) in favor of a given model being the most parsimonious among the candidates. Cum.Wt. = Cumulative Akaike weights. Models: Fish Hue = Fish Richness ~ poly (Hue Diversity, 2): Fish Hue Coral = Fish Richness ~ Hue Diversity + poly (Coral Richness, 2): Fish Coral = Fish Richness ~ Coral Richness.

| Parameter | K | AICc | Delta AICc | AICc Wt. | Cum. Wt |
| --- | --- | --- | --- | --- | --- |
| Fish Hue Coral | 5 | 1024.03 | 0 | 0.98 | 0.98 |
| Fish Coral | 3 | 1031.73 | 7.7 | 0.02 | 1 |
| Fish Hue | 4 | 1054.96 | 30.93 | 0 | 1 |

**Table S3.** Extracted and summarized data from eight studies that evaluated reef fish responses to bleaching events that induced colour loss but maintained coral richness and structure. Data from 133 comparisons were extracted from the eight studies. The standardized mean difference (Hedge’s d) and the corresponding variance of each comparison were aggregated according to the parameter evaluated and averaged at the study level. The aggregate dependent effect size and variance for each parameter were then determined by averaging the studies Hedge’s d and variance (within each parameter).

|  | | | |
| --- | --- | --- | --- |
| Parameter | Paper ID | Effect Size | Variance |
| Fish Richness | ^25^ | 0.41363725 | 0.0646382 |
|  | ^26^ | -0.034745 | 0.03695896 |
|  | ^27^ | 0.50223072 | 0.07791474 |
| Fish Abundance | ^28^ | 1.6222688 | 0.30619848 |
|  | ^29^ | 0.5540655 | 0.05210797 |
|  | ^25^ | 0.5324116 | 0.06497037 |
|  | ^26^ | 0.0960594 | 0.02611204 |
|  | ^30^ | 1.1964212 | 0.11873619 |
|  | ^27^ | 0.8930325 | 0.08878655 |
|  | ^31^ | 1.4444897 | 0.23885296 |
| Recruitment | ^32^ | 0.9694317 | 0.44698989 |
|  | ^28^ | 0.5103869 | 0.30084971 |
|  | ^30^ | 0.1487758 | 0.05111908 |
|  | ^27^ | 1.6895749 | 0.33568475 |
| Labridae Abundance | ^26^ | 0.1405016 | 0.02916757 |
| Pseudochromidae Abundance | ^26^ | -0.1941921 | 0.03754656 |
|  | ^29^ | 0.5540655 | 0.05210797 |
| Nemipteridae Abundance | ^26^ | 0.2429658 | 0.0496092 |
| Apogonidae Abundance | ^26^ | 0.2707735 | 0.03732618 |
| Chaetodontidae Abundance | ^26^ | 0.3566241 | 0.05002474 |
| Gobiidae Abundance | ^26^ | 0.06579594 | 0.04927565 |
|  | ^30^ | 1.19642117 | 0.11873619 |
| Pseudochromidae Abundance | ^26^ | -0.1941921 | 0.03754656 |
|  | ^29^ | 0.5540655 | 0.05210797 |

**SI References**

1. Weller, H. I. & Westneat, M. W. Quantitative color profiling of digital images with earth mover’s distance using the R package colordistance. *PeerJ* **7**, e6398 (2019).

2. Stevens, M., Párraga, C. A., Cuthill, I. C., Partridge, J. C. & Troscianko, T. S. Using digital photography to study animal coloration. *Biol. J. Linn. Soc.* **90**, 211–237 (2007).

3. Luscier, J. D., Thompson, W. L., Wilson, J. M., Gorham, B. E. & Dragut, L. D. Using digital photographs and object-based image analysis to estimate percent ground cover in vegetation plots. *Front. Ecol. Environ.* **4**, 408–413 (2006).

4. Miyagi, R. *et al.* Correlation between nuptial colors and visual sensitivities tuned by opsins leads to species richness in sympatric Lake Victoria cichlid fishes. *Mol. Biol. Evol.* **29**, 3281–3296 (2012).

5. Allen, W. L., Baddeley, R., Scott-Samuel, N. E. & Cuthill, I. C. The evolution and function of pattern diversity in snakes. *Behav. Ecol.* **24**, 1237–1250 (2013).

6. Medina, I., Vega-Trejo, R., Wallenius, T., Symonds, M. R. E. & Stuart-Fox, D. From cryptic to colorful: Evolutionary decoupling of larval and adult color in butterflies. *Evol. Lett.* **4**, 34–43 (2020).

7. Hemingson, C. R., Cowman, P. F., Hodge, J. R. & Bellwood, D. R. Colour pattern divergence in reef fish species is rapid and driven by both range overlap and symmetry. *Ecol. Lett.* **22**, 190–199 (2019).

8. Wu, S. *et al.* Artificial intelligence reveals environmental constraints on colour diversity in insects. *Nat. Commun.* **10**, (2019).

9. Van Belleghem, S. M. *et al.* patternize: An R package for quantifying colour pattern variation. *Methods Ecol. Evol.* **9**, 390–398 (2018).

10. Maia, R., Gruson, H., Endler, J. A. & White, T. E. pavo 2: New tools for the spectral and spatial analysis of colour in r. *Methods Ecol. Evol.* **10**, 1097–1107 (2019).

11. Viechtbauer, W. Conducting Meta-Analyses in R with the metafor Package. *J. Stat. Softw.* **36**, (2010).

12. R Core Team. *R: A language and environment for statistical computing*. (R Foundation for Statistical Computing, 2019).

13. Del Re, A. C. & Hoyt, W. T. *MAd: Meta-Analysis with Mean Differences*. (2014).

14. Centore, P. sRGB Centroids for the ISCC-NBS Colour System. *Munsell Colour Sci Paint.* 21 (2016).

15. Froese, R. & Pauly, D. FishBase. www.fishbase.org (2019).

16. Veron, J. E. N., Stafford-Smith., M. G., Turak, E. & DeVantier, L. M. Corals of the World. www.coralsoftheworld.org (2020).

17. Vorobyev, M., Marshall, J., Osorio, D., de Ibarra, N. H. & Menzel, R. Colourful objects through animal eyes. *Color Res. Appl.* **26**, 4 (2001).

18. Troscianko, J. & Stevens, M. Image calibration and analysis toolbox - a free software suite for objectively measuring reflectance, colour and pattern. *Methods Ecol. Evol.* **6**, 1320–1331 (2015).

19. Kohler, K. E. & Gill, S. M. Coral Point Count with Excel extensions (CPCe): A Visual Basic program for the determination of coral and substrate coverage using random point count methodology. *Comput. Geosci.* **32**, 1259–1269 (2006).

20. Siebeck, U. E., Marshall, N. J., Klüter, A. & Hoegh-Guldberg, O. Monitoring coral bleaching using a colour reference card. *Coral Reefs* **25**, 453–460 (2006).

21. Stokes, M. D. & Deane, G. B. Automated processing of coral reef benthic images: Coral reef benthic imaging. *Limnol. Oceanogr. Methods* **7**, 157–168 (2009).

22. Shihavuddin, A. S. M., Gracias, N., Garcia, R., Gleason, A. & Gintert, B. Image-based coral reef classification and thematic mapping. *Remote Sens.* **5**, 1809–1841 (2013).

23. Cresswell, A. K. *et al.* Translating local benthic community structure to national biogenic reef habitat types. *Glob. Ecol. Biogeogr.* **26**, 1112–1125 (2017).

24. Stuart-Smith, R. D., Brown, C. J., Ceccarelli, D. M. & Edgar, G. J. Ecosystem restructuring along the Great Barrier Reef following mass coral bleaching. *Nature* **560**, 92–96 (2018).

25. Yahya, S. A. S. *et al.* Coral bleaching and habitat effects on colonisation of reef fish assemblages: An experimental study. *Estuar. Coast. Shelf Sci.* **94**, 16–23 (2011).

26. Feary, D., Almany, G., Jones, G. & McCormick, M. Coral degradation and the structure of tropical reef fish communities. *Mar. Ecol. Prog. Ser.* **333**, 243–248 (2007).

27. Feary, D. A., Almany, G. R., McCormick, M. I. & Jones, G. P. Habitat choice, recruitment and the response of coral reef fishes to coral degradation. *Oecologia* **153**, 727–737 (2007).

28. Booth, D. & Beretta, G. Changes in a fish assemblage after a coral bleaching event. *Mar. Ecol. Prog. Ser.* **245**, 205–212 (2002).

29. Coker, D. J., Pratchett, M. S. & Munday, P. L. Coral bleaching and habitat degradation increase susceptibility to predation for coral-dwelling fishes. *Behav. Ecol.* **20**, 1204–1210 (2009).

30. Bonin, M., Munday, P., McCormick, M., Srinivasan, M. & Jones, G. Coral-dwelling fishes resistant to bleaching but not to mortality of host corals. *Mar. Ecol. Prog. Ser.* **394**, 215–222 (2009).

31. Coker, D. J., Pratchett, M. S. & Munday, P. L. Influence of coral bleaching, coral mortality and conspecific aggression on movement and distribution of coral-dwelling fish. *J. Exp. Mar. Biol. Ecol.* **414–415**, 62–68 (2012).

32. McCormick, M. I., Moore, J. A. Y. & Munday, P. L. Influence of habitat degradation on fish replenishment. *Coral Reefs* **29**, 537–546 (2010).
